# IL-1β Signaling Promotes CNS-Intrinsic Immune Control of West Nile Virus Infection

**DOI:** 10.1371/journal.ppat.1003039

**Published:** 2012-11-29

**Authors:** Hilario J. Ramos, Marion C. Lanteri, Gabriele Blahnik, Amina Negash, Mehul S. Suthar, Margaret M. Brassil, Khushbu Sodhi, Piper M. Treuting, Michael P. Busch, Philip J. Norris, Michael Gale

**Affiliations:** 1 Department of Immunology, University of Washington School of Medicine, Seattle, Washington, United States of America; 2 Blood Systems Research Institute, San Francisco, California, United States of America; 3 Department of Comparative Medicine, University of Washington School of Medicine, Seattle, Washington, United States of America; 4 Department of Laboratory Medicine, University of California San Francisco, San Francisco, California, United States of America; 5 Department of Medicine, University of California San Francisco, San Francisco, California, United States of America; 6 Department of Microbiology, University of Washington School of Medicine, Seattle, Washington, United States of America; Yale University School of Medicine, United States of America

## Abstract

West Nile virus (WNV) is an emerging *flavivirus* capable of infecting the central nervous system (CNS) and mediating neuronal cell death and tissue destruction. The processes that promote inflammation and encephalitis within the CNS are important for control of WNV disease but, how inflammatory signaling pathways operate to control CNS infection is not defined. Here, we identify IL-1β signaling and the NLRP3 inflammasome as key host restriction factors involved in viral control and CNS disease associated with WNV infection. Individuals presenting with acute WNV infection displayed elevated levels of IL-1β in their plasma over the course of infection, suggesting a role for IL-1β in WNV immunity. Indeed, we found that in a mouse model of infection, WNV induced the acute production of IL-1β *in vivo*, and that animals lacking the IL-1 receptor or components involved in inflammasome signaling complex exhibited increased susceptibility to WNV pathogenesis. This outcome associated with increased accumulation of virus within the CNS but not peripheral tissues and was further associated with altered kinetics and magnitude of inflammation, reduced quality of the effector CD8^+^ T cell response and reduced anti-viral activity within the CNS. Importantly, we found that WNV infection triggers production of IL-1β from cortical neurons. Furthermore, we found that IL-1β signaling synergizes with type I IFN to suppress WNV replication in neurons, thus implicating antiviral activity of IL-1β within neurons and control of virus replication within the CNS. Our studies thus define the NLRP3 inflammasome pathway and IL-1β signaling as key features controlling WNV infection and immunity in the CNS, and reveal a novel role for IL-1β in antiviral action that restricts virus replication in neurons.

## Author Summary

Since its introduction into North America in 1999, West Nile virus (WNV) has emerged as a leading cause of viral encephalitic disease in the United States. While low level inflammation is important for clearance of WNV, high levels of inflammation are associated with increased disease. The goal of this study was to identify host signaling pathways that control the balance of inflammation and protective immunity to WNV. Using a mouse model of infection, we identified a central nervous system (CNS)-intrinsic requirement for the NLRP3 inflammasome and IL-1β signaling in limiting WNV associated disease within the CNS. First, IL-1β signaling was essential for regulating the magnitude and kinetics of inflammation within CNS. Secondly, the absence of IL-1β signaling disrupted the quality of the effector T lymphocyte response against the virus. Finally, these dysregulated immune responses were linked to a direct ability for IL-1β signaling to synergize with type I IFN signaling and limit virus replication within cortical neurons, key target cells of WNV infection within the CNS. Together this study identifies the NLRP3 inflammasome and IL-1β signaling as key restriction factors that act to regulate viral load and the quality of inflammatory responses within the CNS to impart protective immunity against WNV infection.

## Introduction

West Nile virus (WNV) is a single stranded, positive sense RNA virus of the *flaviviridae* family, and is a prototypical *Flavivirus* related to Yellow fever virus, Tick borne encephalitis virus, Japanese encephalitis virus (JEV) and Dengue virus [1]–[2], all of which are major public health threats. Among these viruses, WNV has emerged into the Western hemisphere and continues its spread through into North America [3]. WNV is normally maintained in mosquito and avian reservoirs, with infection of human and other animals occurring through contact with infected mosquitoes [4]–[5]. Infection is largely controlled acutely; however WNV can spread to the central nervous system (CNS), leading to encephalitic disease and death [6]–[7]. Overall however, the immune processes within the CNS that serve to control WNV infection and pathogenesis are not well defined.

WNV pathogenesis has been studied in murine models of infection to show that the virus initially replicates in epithelial cells and skin Langerhans dendritic cells (DCs) at the site of mosquito inoculation [8]. The virus then traffics to the draining lymph node, leading to secondary viremia and infection of the spleen where it can replicate in macrophage and DC subsets [5], [9]. After amplification in peripheral tissues, WNV spreads to the CNS, where it replicates in neurons, causes neuronal destruction, and imparts inflammation leading to encephalitis that is comparable to human disease [5]–[7]. Both innate and adaptive immune defenses serve to control tissue tropism and initial spread of virus into to the CNS [10]–[13], while T lymphocyte responses are involved in mediating clearance of virus following CNS entry [14]–[15]. In particular, CD8^+^ T cells are thought to be the main contributors to late CNS clearance of WNV through mechanisms involving IFN-γ, TNF-α and perforin [13]–[14], [16].

The inflammatory response is a key component in protective immunity against WNV infection. However, this response must be kept in check to limit bystander destruction of both peripheral and CNS tissues. For example encephalitis, which is marked by inflammatory cell recruitment to the CNS, has been shown to be both protective as well as destructive to CNS tissue during WNV infection [17]. Recruitment of populations of peripheral CD45^+^ leukocytes into the CNS has been shown to be important for limiting WNV pathogenesis [18]–[20]. In contrast, in other studies CD45^+^ leukocytes were shown to enhance susceptibility to WNV, likely due to increased immune-pathology associated with inflammatory cell-mediated destruction of CNS tissue. Thus, while inflammation is required for clearance of WNV in the CNS, the timing and magnitude of this inflammatory response must be tightly regulated to avoid off target pathology.

IL-1 signaling is involved in multiple aspects of the immune response to infection including immune regulation of inflammation, modulation of adaptive immune programs and direct antiviral control of pathogens [21]–[24]. These processes are driven by IL-1α and IL-1β which signal through the IL-1R1 (IL-1Rα) and MyD88 to drive downstream NF-κB activation and subsequent expression of genes whose products regulate the immune response to infection [21], [25]–[26]. Activation of IL-1 requires two distinct signals, “signal 1” which drives mRNA expression in an NF-κB-dependent manner and “signal 2” which processes the cytokine to its functional form [27]. “Signal 2” processing of IL-1β to its active form is mediated by inflammasomes, signaling structures comprised of NOD-like receptors (NLRs), adaptor molecules such as ASC and the effector, Caspase-1 [27]–[28]. In contrast, IL-1α is not processed by the inflammasome and is instead thought to processed and activated by other host protease pathways [29] suggesting that the majority of immune responses driven by inflammasome activation are mediated by IL-1β and not IL-1α.

Several distinct inflammasomes have been described based upon their inclusion of a specific NLR or signaling-initiator molecule, including the NLRP1, NLRC4, NLRP3, RIG-I and AIM2 [30]. Of these, the NLRP3 inflammasome has been the best characterized to mediate IL-1β secretion in response to RNA viruses *in vitro* and *in vivo* [24], [31]. Inflammasome activation and IL-1β signaling are important for immunity against several viruses including Influenza A, hepatitis B, Sendai and vesicular stomatitis virus (VSV) [24] and drive host responses that regulate cellular infiltration to sites of infection [32]–[35], adaptive immunity [23], [36] and direct viral control in combination with other host factors such as IFN-α/β (type I IFN), IFN-γ and TNF-α [21]–[22], [37]. In the context of CNS infection, IL-1 signaling has been associated with both protection and enhancement of disease. The synergistic effects of IL-1β and TNF-α were associated with protection against encephalitis by the neurotropic virus HSV-1 [35] while IL-1β^−/−^ animals showed increased pathogenesis and lethality to Sindbis virus [38]–[39]. Thus, IL-1 signaling likely functions in a context-dependent manner to control or exacerbate disease. Little is known about IL-1 signaling in immunity to *flavivirus* infection. Recently, JEV was shown to trigger IL-1β secretion from astrocytes and microglia [40], and this was subsequently shown to be dependent on the NLRP3 inflammasome [41]. In addition, IL-1β expression was shown to be important for recruitment of DCs to the lymph node after WNV infection [8]. Thus, inflammasome signaling may integrate with multiple immune pathways to participate in the control of *Flavivirus* infection.

In this study, we systemically examined the role of IL-1 signaling in WNV infection to show that IL-1β signaling driven by the NLRP3 inflammasome acts to mediate protective immunity against infection. We reveal a novel role for IL-1β in specifically limiting viral replication within the CNS. Mechanistically, IL-1β synergizes with type I IFN to control virus replication. Moreover, we found that the lack of viral control in *Il-1r^−/−^* mice correlated with defects in CNS inflammation, T lymphocyte effector activity, and neuropathology. Our observations link the production of IL-1β in the CNS to restriction of WNV replication in neurons and limitation of CNS disease and thus we conclude that the NLRP3 inflammasome and IL-1 signaling are important determinants of immune regulation that impart protective CNS inflammation and control of WNV infection.

## Results

### IL-1β production associates with human WNV infection

In order to assess whether IL-1 was associated with human WNV infection, we examined cytokine expression in the plasma from individuals infected with WNV. Blood donors, testing positive for WNV RNA after routine blood screening were enrolled in a follow-up study to assess their plasma cytokine levels over a six-month period after their initial blood donation (index). We observed that the levels of IFN-γ and TNF-α, immune factors important in immunity to WNV, [13], [18] were enhanced in individuals infected with WNV and the expression of these factors was maintained for long periods of time in these individuals (Figure S1A,B).

**Figure 1:**
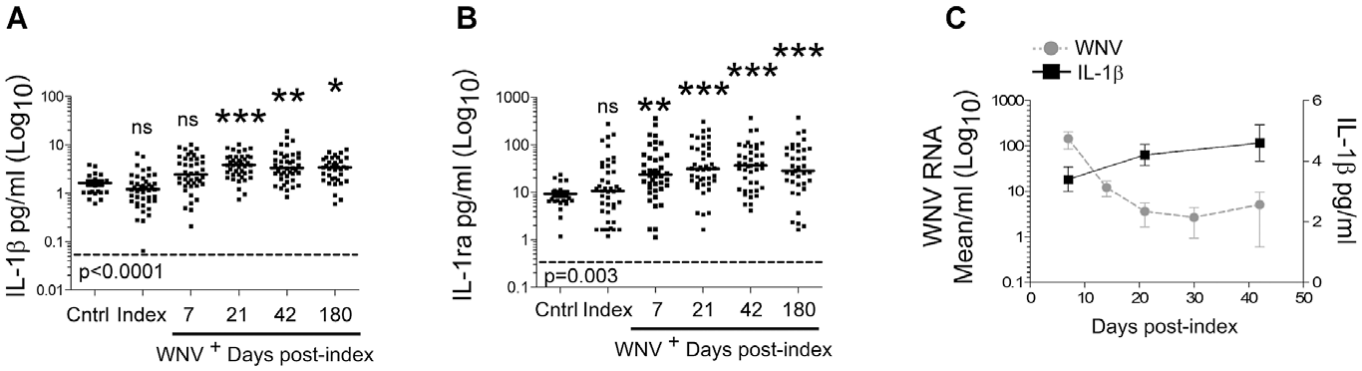
IL-1β is produced during acute West Nile Virus infection in humans. IL-1 in plasma of WNV infected (n = 43) and control subjects (n = 21). Plasma from blood donors testing positive for WNV RNA was collected at time-points from 7 to 180 days post-Index and was compared to control (cntrl) samples. Samples were then assessed for IL-1β (**A**) or IL-1ra (**B**). IL-1β correlates inversely with WNV load. The level of WNV RNA was measured by real-time RT-PCR for days 7, 14, 21, 30, and 42 post-index (**C, left axis**). Levels of IL-1β at days 7, 21, and 42 post-index are displayed (**C, right axis**). Middle bars represent the median for each group. * p<0.05, ** p<0.005, *** p<0.0005 values are reflective of significance compared to control samples. ns refers to not-significant. P value-inset (p<0.0001 (**A**), p = 0.003 (**B**)) is reflective of trend analysis comparing the trend in WNV^+^ blood donors over time post-infection. Dashed lines represent the minimum detectable concentration of the assay.

In addition to these known host restriction factors, the levels of IL-1β were enhanced in the plasma of WNV^+^ individuals and displayed significant increase over time (trend analysis) at 7, 21, 42, and 180 days post-index when compared to normal controls (Cntrl) (Figure 1A; trend analysis (p<0.0001), inset). This was similar to the expression of the IL-1r antagonist (IL-1ra), a natural regulator of IL-1β [25], which is known to track with expression of the cytokine (Figure 1B; trend analysis (p = 0.003), inset). In contrast, levels of IL-1α in the plasma were not altered by WNV infection at any time-point tested (Figure S1C). These data are consistent with the expression of IFN-γ and TNF-α, and is in line with recent data demonstrating that WNV RNA persists at low levels out to 200 days post-index in whole blood of infected humans [42] and *in vivo* in peripheral tissues of mice [43] for extended periods of time.

We found that WNV RNA levels decreased from day 7 to day 21 post-index (Figure 1C). This correlated with the induction of IL-1β at day 7, suggesting that initial viral load might drive the expression of the cytokine. In contrast, an inverse correlation was found between the levels of WNV viral load and the levels of IL-1β in plasma during the first six weeks post-index (GEE estimate = −1.28, p = 0.01). Together, these data demonstrate that IL-1β expression but not IL-1α expression is associated with WNV infection in humans and indicates that WNV infection triggers the inflammasome signaling pathway to induce IL-1β during infection.

### Inflammasome and IL-1β signaling are important for immunity to West Nile virus *in vivo*

**Figure 2:**
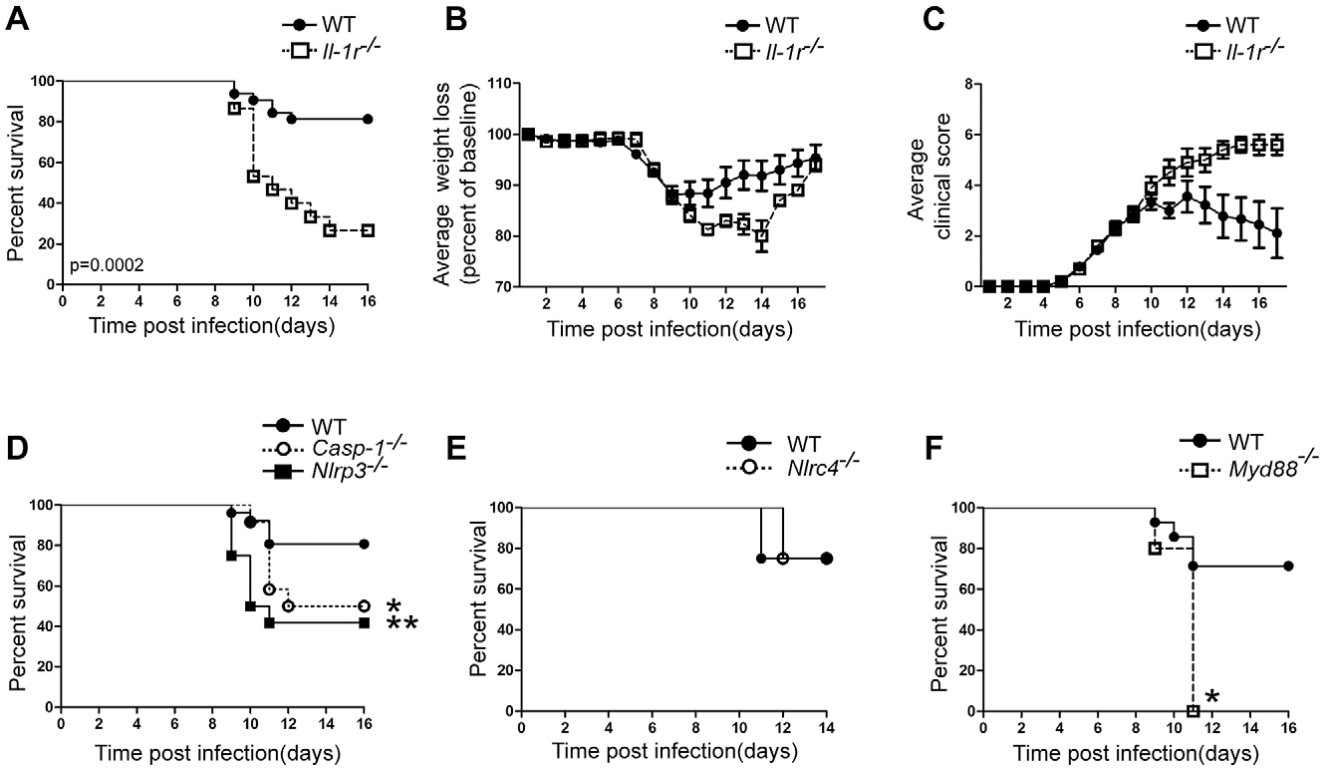
IL-1β signaling and the NRLP3 inflammasome are required for immunity against WNV. 6–10 wk old age matched WT (closed circles; n = 26) or *Il-1r^−/−^* (open squares; n = 14) (**A**) or *Caspase-1^−/−^* (open circles; n = 12) and *Nlrp3^−/−^* (closed square; n = 12) (**D**) or WT (closed circles, n = 5) and *Nlrc4^−/−^* (open circles; n = 4) (**E**) or WT (closed circles, n = 5) and *Myd88^−/−^* (closed square; n = 5) (**F**) animals were infected with 100 PFU WNV-TX via s.c. foot-pad inoculation and monitored for survival over the course of 14–16 days (**A, D–F**). Infected WT (closed circles) or *Il-1r^−/−^* (open squares) animals from panel A, were monitored daily for weight loss (**B**) or scored for hind limb paralysis and morbidity (**C**) to day 16 post infection. *p<0.05, ** p<0.005, *** p<0.0005.

To evaluate the role of IL-1β signaling in immunity against WNV infection, we assessed infection in a well characterized murine model of WNV subcutaneous (s.c) footpad inoculation [5], [9]. C57BL/6-WT (WT) or mice deficient in their ability to respond to IL-1α or IL-1β, IL-1Rα chain^−/−^ (*Il-1r^−/−^*), were challenged with 10^2^ plaque-forming units (PFU) of a virulent strain of WNV, WNV-TX [44]. *Il-1r^−/−^* mice were found to be highly susceptible to WNV infection (Figure 2A) displaying increased mortality (73.3% mortality compared to 23% in WT mice). WT mice presented with clinical signs of disease, including mild-paresis and weight loss, by day 6 post-infection (p.i.) with WNV. These responses peaked by day 10 p.i. and the majority of mice were fully recovered by day 16 p.i. (Figures 2B,C). While Il-*1r^−/−^* mice displayed identical onset of clinical disease and weight loss as compared to WT mice, they presented with enhanced disease/hind limb dysfunction (clinical scoring), weight loss and eventual death by day 10 p.i. (Figures 2A,B,C), thus demonstrating an important role for this signaling pathway in preventing late stage WNV-mediated disease.

IL-1β, but not IL-1α, requires processing via the NLRP3-inflammasome for its functional secretion and activity against multiple viruses [29]. Therefore we next examined whether NLRP3 signaling was responsible for the observed phenotype in *Il-1r^−/−^* animals infected with WNV. We found that mice lacking NLRP3 (58.3% mortality) or its downstream effector, Caspase-1 (50% mortality), displayed increased susceptibility and clinical disease in response to WNV challenge (Figure 2D, S2A–D). This outcome was in contrast to infection of mice lacking NLRC4, which mediates a distinct inflammasome not associated with IL-1β processing in response to viral infection [24]. In these mice, the absence of NLRC4 did not influence susceptibility (Figure 2E) or clinical disease (Figures S2E,F) to WNV infection compared with WT controls. Thus, these observations are consistent with WNV infection triggering “signal 2” processing of IL-1β *in vivo* through the NLRP3 inflammasome to mediate immunity. In addition to inflammasome processing of IL-1β, we also examined the requirement for Myd88, the adaptor protein that propagates both IL-1 and TLR signaling [26]. Similar to our observations in *Il-1r^−/−^* and NLRP3-inflammasome deficient animals, *Myd88^−/−^* animals were highly susceptible to WNV challenge (100% mortality) (Figure 2F). These results correlate with recent observations in mice deficient in MyD88, which display enhanced mortality to WNV due to lack of CNS-specific control of the virus [19] and likely reflects the essential role of MyD88 in signaling the response to IL-1β [25]–[26]. Together, these data are consistent with a model in which IL-1β activation via the NLRP3 inflammasome and signaling via MyD88 act as the major pathways involved in mediating the *Il-1r^−/−^* phenotype to control late stage WNV disease.

### IL-1β responses control West Nile virus replication within the CNS

**Figure 3:**
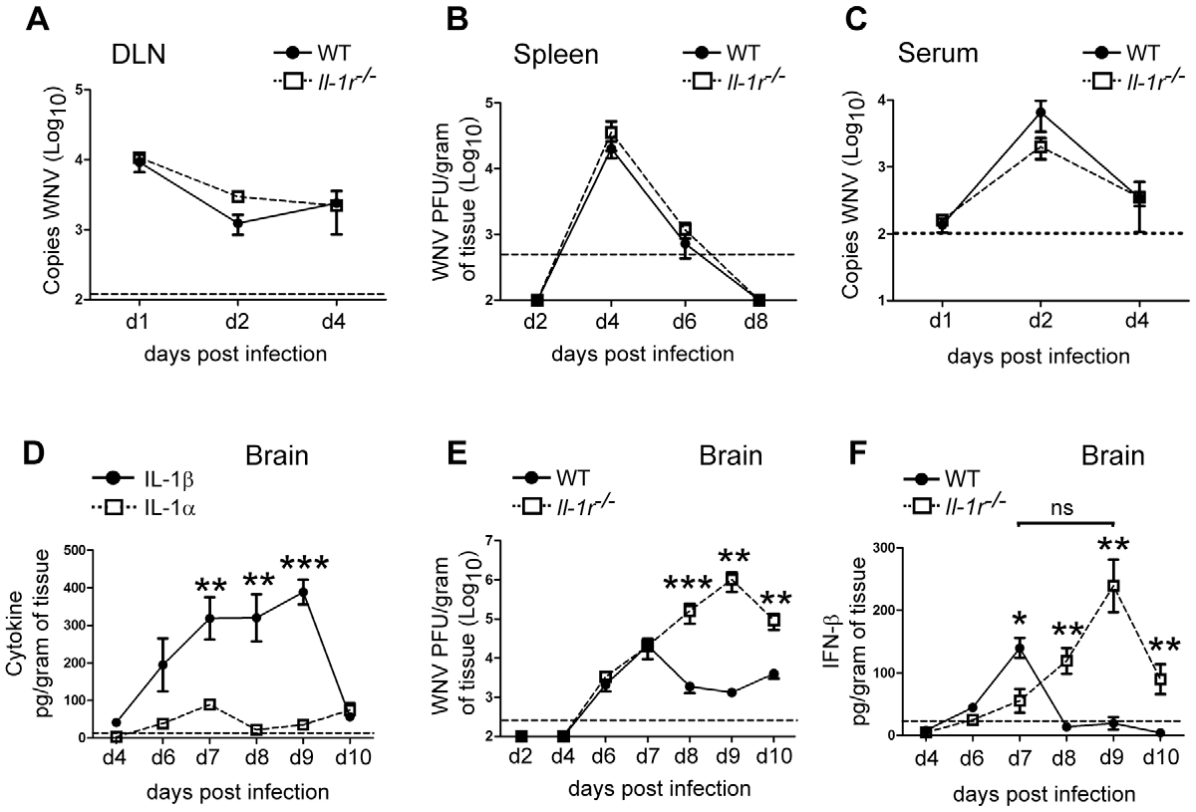
IL-1β signaling is required for CNS-specific protective immunity against WNV. Examination of *in vivo* IL-1 cytokine expression and viral load in WT and *Il-1r^−/−^* animals. 6–10 wk old WT mice were infected s.c. with 100 PFU WNV-TX02 or mock infected. Viral loads were assessed from WT (closed circles) or *Il-1r^−/−^* mice (open squares) by plaque assay from spleen (**B**) and brain (**E**) lysates or by Taqman based qRT-PCR using specific primers and probes to WNV for DLN (**A**) and Serum (**C**). Cytokine expression for IL-1α and IL-1β was assessed by Luminex array for Brain lysates (**D**) or IFN-β levels by ELISA (**F**) Data are shown as the mean +/− S.E.M. for n = 3–6 mice per time-point. *p<0.05, *** p<0.0005. Dashed lines represent the lower limit of detection for each assay. BLD denotes below limit of detection.

In order to more fully understand the contribution of IL-1 signaling in the *in vivo* response to WNV, we examined IL-1α and IL-1β expression in tissues known to be active sites of viral replication in WT mice [5], [9]. WNV infection induced variable expression of both IL-1α and IL-1β in the draining popliteal lymph node (DLN) and spleen of infected mice (Figures S3A,B) however neither cytokine were detected in the serum of infected WT animals at any time-point tested (data not shown). In contrast to these peripheral tissues, we found the most dramatic differences in IL-1α and IL-1β in the brains of infected animals. Here, IL-1β reached detectable levels by day 6 p.i. and was maintained at high levels through day 9 p.i. when the cytokine level peaked (Figure 3D). This was in contrast to IL-1α which was expressed only at low levels throughout the course of infection (Figure 3D) suggesting that IL-1β may play the predominant role in immunity against WNV. We observed no difference in the magnitude of IL-1α (data not shown) or IL-1β (Figure S3C) levels in the CNS between WT and *Il-1r^−/−^* animals.

We next assessed whether the tissue-specific expression of IL-1β influenced WNV replication within peripheral organs, blood, and the CNS. We observed no difference in the kinetics or magnitude of viral load detected between WT and *Il-1r^−/−^* deficient animals within the spleen or serum (Figures 3B,C), and this was consistent with the lack of appreciable IL-1β at the peak of viral replication in these tissues (Figures S2B, data not shown). In contrast, we observed a modest trend of increased viral load at day 2 post infection in the DLN (Figure 3A), suggesting a role for IL-1β signaling in immunity to WNV at early time-points. Despite only minimal differences in viral load in peripheral organs, viral load in the brains of *Il-1r^−/−^* mice was increased when compared to WT mice as early as day 8 p.i. and this persisted through day 10 p.i. (Figure 3E). A similar enhancement of viral load was observed in the brains of MyD88 and NLRP3-deficient animals (Figures S4A,B), as well as, in day 10 spinal cords of all IL-1 signaling-deficient animals (Figures S4C). The difference in viral control were not due to earlier entry of virus in the CNS as virus was first detected in the CNS at day 6 p.i. with similar magnitude of viral load in both strains (Figure 3E). Instead, the initial differences in viral control occurred between day 7 and 8 p.i. and correlated to a time-point in which IL-1β was detected at high levels in WT mice (Figure 3D,E). Together, these data suggest NLRP3 inflammasome activation of IL-1β and the subsequent actions of IL-1β operate to limit the replication and/or spread of WNV within the CNS.

Type I interferon (IFN) signaling has been shown to contribute to control of tissue tropism and CNS control of WNV [9], [11]–[12]. Therefore, we tested whether type I IFN could be involved in the lack of viral control in the DLN and CNS of inflammasome-deficient animals. We did not observe a significant difference in secretion of IFN-β in the serum or spleen of infected *Il-1r^−/−^* or WT animals (data not shown). In contrast, we did observe a significant reduction in IFN-β expression in the DLN at day 2 p.i. in *Il-1r^−/−^* mice as compared to their WT counterparts (Figure S3D). This difference correlated to a trend of increased viral load (Figure 3A) but did not appreciably impact overall peripheral virus replication (Figures 3B,C). In contrast to peripheral tissues, we observed differential expression of IFN-β within the CNS between WT and *Il-1r^−/−^* animals. WT mice displayed early IFN-β expression within the CNS with peak levels at day 7 p.i. (Figure 3F). This was consistent with the peak of viral load in these mice suggesting a direct correlation between virus replication and type I IFN production. In contrast, *Il-1r^−/−^* animals showed a delay in expression of IFN-β, with levels tracking with maximal viral load and peaking at day 9 p.i. (Figure 3F). Importantly, the magnitude of the peak of IFN-β expression was not significantly altered between WT and *Il-1r^−/−^* animals despite significantly higher viral loads in the absence of IL-1β signal (Figures 3E,F). These observations suggest that sustained IL-1β signaling in the CNS is important in maintaining efficient activity and/or early type I IFN signaling that controls WNV infection.

### IL-1β signaling regulates the CNS inflammatory response during West Nile virus infection

IL-1β signaling has been associated with various immune functions including regulation of cell recruitment and inflammation, modulation of adaptive immunity and direct anti-viral activity. Therefore we assessed these activities as possible mechanisms by which IL-1β mediated WNV control in the CNS. We first examined CNS infiltration of total CD45^+^ leukocytes as well as a subpopulation of CD45^+^CD11b^+^ cells (a population which is comprised of macrophage dendritic cell and neutrophil subsets) as these cell populations have been previously associated with immunity against WNV in the CNS [18]–[20]. Brains of WT or *Il-1r^−/−^* mice were harvested at day 6, 7, 8 and 10 p.i. with WNV and the kinetics of CD45^+^ leukocyte infiltration were assessed by flow cytometry.

**Figure 4:**
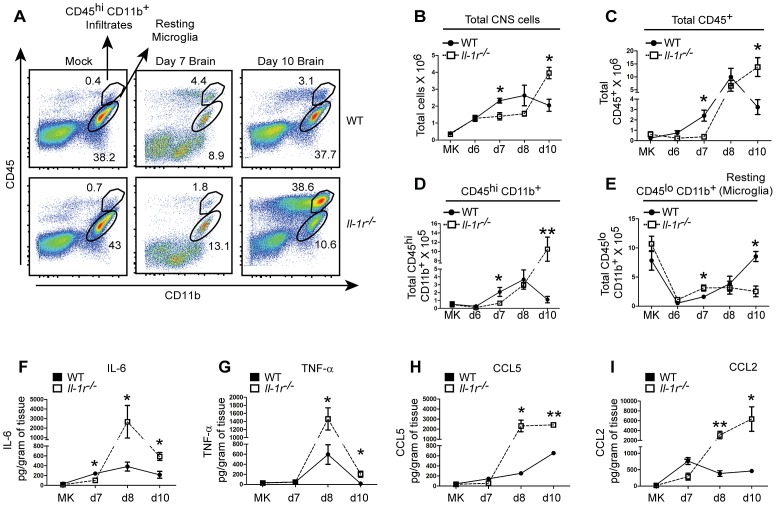
IL-1β signaling is critical for regulating inflammation in the CNS during WNV infection. Assessment of inflammatory responses in the CNS of WT (closed squares) or *Il-1r^−/−^* (open squares) mice. Leukocyte infiltration into the CNS was assessed by flow cytometry at days 6–10 post infection (p.i.) with WNV-TX (**A–E**) as derived from total cell numbers. Cells recovered from perfused whole brain were stained with antibodies to CD45 and CD11b and total cell number (**B–E**) was assessed by flow cytometry. Representative dot-plot analysis for CD11b and CD45 infiltrates and microglia at mock, day 7 and day 10 p.i. is shown (**A**). Brains were harvested from tissues of WNV-TX02 infected mice at indicated time-points homogenized and assessed for cytokine and chemokine expression by luminex array (**F–I**). Data are shown as mean +/− S.E.M. for n = 3–4 per time-point and are compiled from 2–3 independent experiments. * p<0.05, **p<0.005.

We observed minimal infiltration of CD45^+^ leukocytes in the brains of WT or *Il-1r^−/−^* mice at day 6 p.i.(Figures 4C). However, by day 7 p.i., the numbers of total cellular infiltrates, (Figure 4B), as well as the frequency and total numbers of CD45^+^ leukocytes (Figures S5A, 4C) and CD11b^+^/CD45^+^ infiltrates (Figures S5B, 4A,D) was increased in WT animals. This was followed by a peak in accumulation of cell infiltrates at day 8 p.i. and subsequent reduction in cell numbers by day 10 p.i. (Figures S5A,B, 4A–C) which tracked with parallel kinetics of viral load in the CNS of WT mice (Figure 3E). Conversely, *Il-1r^−/−^* mice displayed reduced numbers total cell infiltrates (Figure 4B) as well as frequency and total numbers of CD45^+^ leukocytes (Figures S5A, 4C) and CD11b^+^/CD45^+^ (Figures S5B, 4A,D) at day 7 p.i in the CNS. Furthermore, despite reaching levels of infiltrates comparable to WT mice at day 8 p.i., *Il-1r^−/−^* animals showed a continued increase in immune cell infiltrates through day 10 p.i., suggesting that the magnitude of inflammation was directly correlated to viral load (Figure 3E) in the CNS in these animals.

Resting microglia express CD11b but are distinguished from activated microglia or infiltrating myeloid cells by lower levels of CD45 expression. We observed the numbers of resting microglia, (CD11b^+^/CD45^lo^), were reduced in the CNS at early times (day 6–7p.i.) in WT and *Il-1r^−/−^* animals (Figures 4E, S5C) suggesting increased activation of a subset of microglia upon viral entry into the CNS in both strains of mice. However, while the numbers of non-activated microglial cells was restored to basal levels by day 10 p.i. in WT mice they remained predominantly active day 10 p.i. in *Il-1r^−/−^* animals further suggesting that lack of IL-1β signaling imposes a disruption in CNS inflammatory homeostasis during WNV infection (Figures 4A,E, S5C).

Pro-inflammatory cytokines (TNF-α, IL-6), and chemokines (CCL5, CCL2) which are involved in the recruitment of CD45^+^ leukocytes, were also enhanced in the CNS of *Il-1r^−/−^* animals at late time-points when compared to WT controls (Figures 4F–I) implying that the increase in these inflammatory mediators likely contributes to the increased inflammation within the CNS of *Il-1r^−/−^* mice. In contrast, IL-6 (Figure 4F) and CCL2 (Figure 4I) were expressed at comparably lower levels at day 7 p.i. in the CNS of *Il-1r^−/−^* animals and associated with reduced infiltration of peripheral inflammatory leukocytes. Interestingly we observed increased inflammatory responses in the CNS of NLRP3-deficient animals, which displayed high CNS viral load and enhancement of IL-6, TNF-α and CCL5 levels after WNV infection (Figures S5D–F). Together, these observations indicate that the NLRP3-inflammasome and IL-1β signaling act early during WNV infection to mediate efficient CNS recruitment of immune cells and control CNS viral load.

### Loss of IL-1β signaling leads to increased neuropathology in the CNS of West Nile virus infected animals

**Figure 5:**
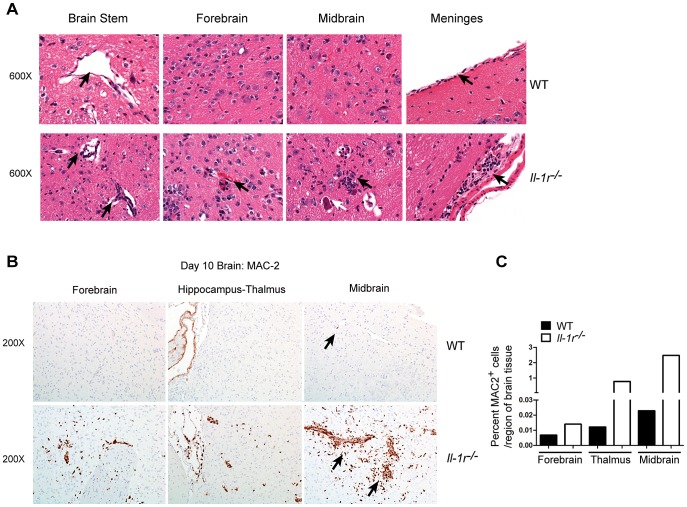
IL-1β signaling is important for limiting neuropathology within the CNS. Histological analysis from day 10 p.i. sagittally cut brain sections from paraffin embedded tissue. Representative formalin-fixed hematoxylin and eosin-stained sections of day 10 p.i. brains from WT (top panels) and *Il-1r−/−* (bottom panels) sections (**A**). Brain areas as indicated in figure. Original magnification 600×. In all regions of the WT brain, the sections are histologically unremarkable whereas lesions were readily apparent in the *Il1r* ^−/−^ tissues. Brainstem: black arrows indicate perivascular regions; note the mildly increased cellularity in the *Il1r* ^−/−^ vessel wall and immediate perivascular space. Forebrain: there is a mild focus of acute perivascular hemorrhage (arrow). Midbrain: There is a focus of mild inflammation associated with shrunken and eosinophilic neuron suggestive of neuronal degeneration (white arrow) which was in association with inflammatory infiltrates. Meninges: Black arrow indicates expansion of the meninges with moderate inflammatory infiltrates; compare to WT which is histologically unremarkable. Data are representative of two animals per genotype. (**B**) Representative day 10 p.i. Mac-2 immunohistochemical stained sections (brain regions are as indicated in the figure) in WT (top panel) or *Il-1r^−/−^* (bottom panel) sections. Positive signal is as indicated by brown staining; original magnification for all panels, 200×. Mac-2 ^+^ cells are present perivascularly (black arrows) and in the adjacent parenchyma (Non-specific staining of the choroid is present (WT top panel hippocampus-thalamus) and *Il-1r^−/−^* (data not shown). (**C**) Quantification of MAC-2 signal to tissue ratio.

Regulated inflammation in the brain is an important component of the immune clearance of neurotropic viral pathogens [17]. This process is consistent with the regulated CNS inflammation observed in WNV-infected WT mice (peak inflammatory CNS response at day 8 which was reduced by day 10 p.i. (Figure 4). Thus, in the absence of IL-1β signaling, the enhanced inflammation observed in *Il-1r^−/−^* mice might actually be detrimental to the host. Indeed, hematoxylin and eosin (H&E) stained histological sections of brain tissue from *Il-1r^−/−^* animals revealed encephalitis at day 10 p.i. marked by perivascular cuffing and inflamed meninges (Figure 5A; black arrows, compare top and bottom panels). Tissue damage was associated with multiple regions of the CNS and included edema and hemorrhage (Figure 5A; Forebrain), mononuclear meningitis (Figure 5A; meninges) and neuronal dropout (Figure 5A-Midbrain; white arrows)

We also observed enhanced macrophage staining (MAC-2) throughout the CNS tissue of WNV-infected, *Il-1r^−/−^*, mice at day 10 p.i. (Figure 5B). Increased accumulation of MAC-2^+^ cells was observed in the forebrain and hippocampus-thalamus but was most evident in the midbrain, where we observed a 22-fold increase in MAC-2 expression in *Il-1r^−/−^* compared to WT controls (Figure 5B,C). This expression was most prominent in and around blood vessels (Figure 5B; black arrows), and within the parenchyma of *Il-1r^−/−^* but not WT brain tissue sections (Figure 5B, bottom panel). This pattern of staining was comparable to the regions of immune infiltrates identified by H&E staining (Figure 5A) and suggests that increased infiltration of macrophage subsets is associated with the tissue damage observed in the CNS of *Il-1r−/−* animals. Thus we conclude IL-1β-dependent control of WNV in the CNS regulates the magnitude of inflammation during acute infection to reduce tissue damage.

### IL-1β signaling is required for proper T lymphocyte effector activity in the CNS of West Nile virus infected mice

**Figure 6:**
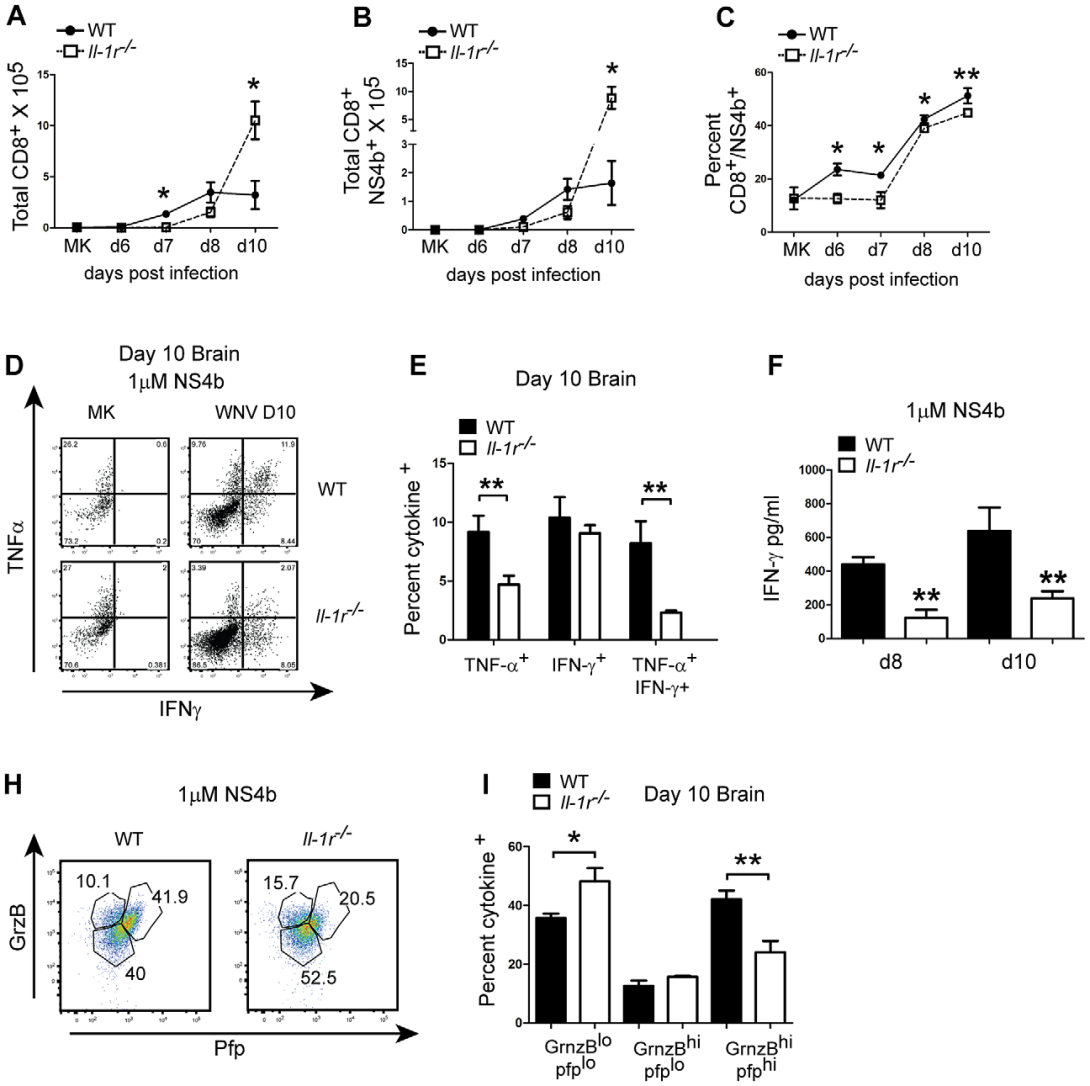
IL-1β signaling is critical for regulating CD8^+^ T cell effector activity within the CNS. Examination of T lymphocyte effector activity in the CNS by flow cytometry. Cell were isolated from the brains of mock or day 6–10 p.i. WT (closed circles) or *Il-1r^−/−^*(open squares) and staining for CD3, CD8 and WNV-NS4b tetramer was performed. Quantitation of CD3^+^/CD8+ T lymphocytes (**A**), CD3^+^/CD8^+^/NS4b^+^ (**B**) as derived from total cell numbers or frequency of CD3^+^/CD8^+^/NS4b^+^ cells (**C**). Brains were harvested from day 8 or 10 p.i. and stimulated with 1 µM WNV-NS4b for 4 hrs (**D,E, H,I**) and assessed for cytokine expression by intracellular staining. Representative dot-plot analysis for IFN-γ and TNF-α expression at day 10 p.i. in the brain (**D**) or perforin and granzyme B (**H**). Data are shown as percent cytokine positive for WT (closed box) or *Il-1r^−/−^* (open box) (**E,I**). Quantification of IFN-γ secretion by ELISA. Total brain cells (2.5e5) were stimulated with NS4b for 24 hrs and IFN-γ was quantified by ELISA for WT (closed circles) and *Il-1r^−/−^* (open squares) cells (**F**). Data are shown as mean +/− S.E.M. for n = 3–4 mice per time-point (**A–E, H,I**) or n = 3–7 (**F**) and are representative of 2–3 independent experiments. *p<0.05, ** p<0.005.

CD8^+^ T cell responses are important for clearing WNV infection from the CNS [14]–[16]. We therefore assessed: 1) total Ag-specific cell accumulation, 2) cytokine secretion properties and 3) cytolytic properties of CD8^+^ T cells within the CNS of WT and *Il-1r^−/−^* mice. Similar to infiltrating leukocytes, the initial entry of total CD8^+^ (Figures 6A) as well as WNV-NS4b-restricted (antigen-specific) T cells (Figures 6B) into the CNS was delayed in *Il-1r^−/−^* animals when compared to WT mice. However, by day 10 p.i. total CD8^+^ (Figure 6A), CD4^+^ (data not shown) and antigen-specific CD8^+^ T cells (Figure 6B), were enhanced in *Il-1r^−/−^* animals, further demonstrating a breakdown in the regulation of inflammation. Interestingly, although the total number of antigen-specific cells was increased in the absence of IL-1β signaling, the frequency of these cells within the CD8^+^ T cell pool was reduced at each time-point tested compared to wild-type mice (Figure 6C), thus suggesting that effector activity in the CNS might be altered in these mice.

In order to test this, total cells were recovered from the CNS at day 8 and day 10 p.i. and stimulated *ex vivo* with the immunodominant T cell epitope peptide-WNV-NS4b (NS4b). We did not observe any differences between *Il-1r^−/−^* and WT cells in their frequency of cytokine production at day 8 p.i. (data not shown). However, by day 10 p.i., the frequency of TNF-α^+^/IFN-γ^+^ double positive and TNF-α^+^, single cytokine producing cells were significantly lower in *Il-1r^−/−^* cells when compared to WT infected mice (Figures 6D,E). In contrast, the frequency of IFN-γ^+^ single cytokine expressing CD8^+^ T cells were comparable between the two genotypes, demonstrating an overall loss in multi-functionality but not complete dysfunction in *Il-1r^−/−^* CD8^+^ T cells (Figures, D,E). In addition, restimulation of equal numbers of WT or *Il-1r^−/−^* total brain cells (2.5e^5^) with the NS4b peptide promoted significantly less IFN-γ production at both time-points tested (Figure 6F) consistent with the reduced frequency of antigen-specific cells at these time-points (Figure 6C). Cytolytic activity was also found to be compromised in *Il-1r^−/−^* T cells, as defined by reduced frequency of cells expressing perforin and this was accompanied by a significant increase in granzyme B/perforin low cells by day 10 p.i. (Figure 6H,I). Taken together these data serve to link dysregulated inflammation in the CNS of *Il-1r^−/−^* mice with defective antigen-specific CD8^+^ T cell effector cytokine and cytolytic activity in the CNS.

Despite a requirement for IL-1β signaling in regulating the T lymphocyte response, humoral immunity remained relatively intact as we observed no differences in the levels of WNV-specific serum IgM and IgG or neutralizing activity of these antibodies between WT and *Il-1r^−/−^* deficient animals (Table S1). Thus, IL-1β signaling is important for maintaining proper T but not B lymphocyte effector activity during WNV infection.

### IL-1β signaling acts in an antiviral manner *in vivo* to control West Nile virus within the CNS

**Figure 7:**
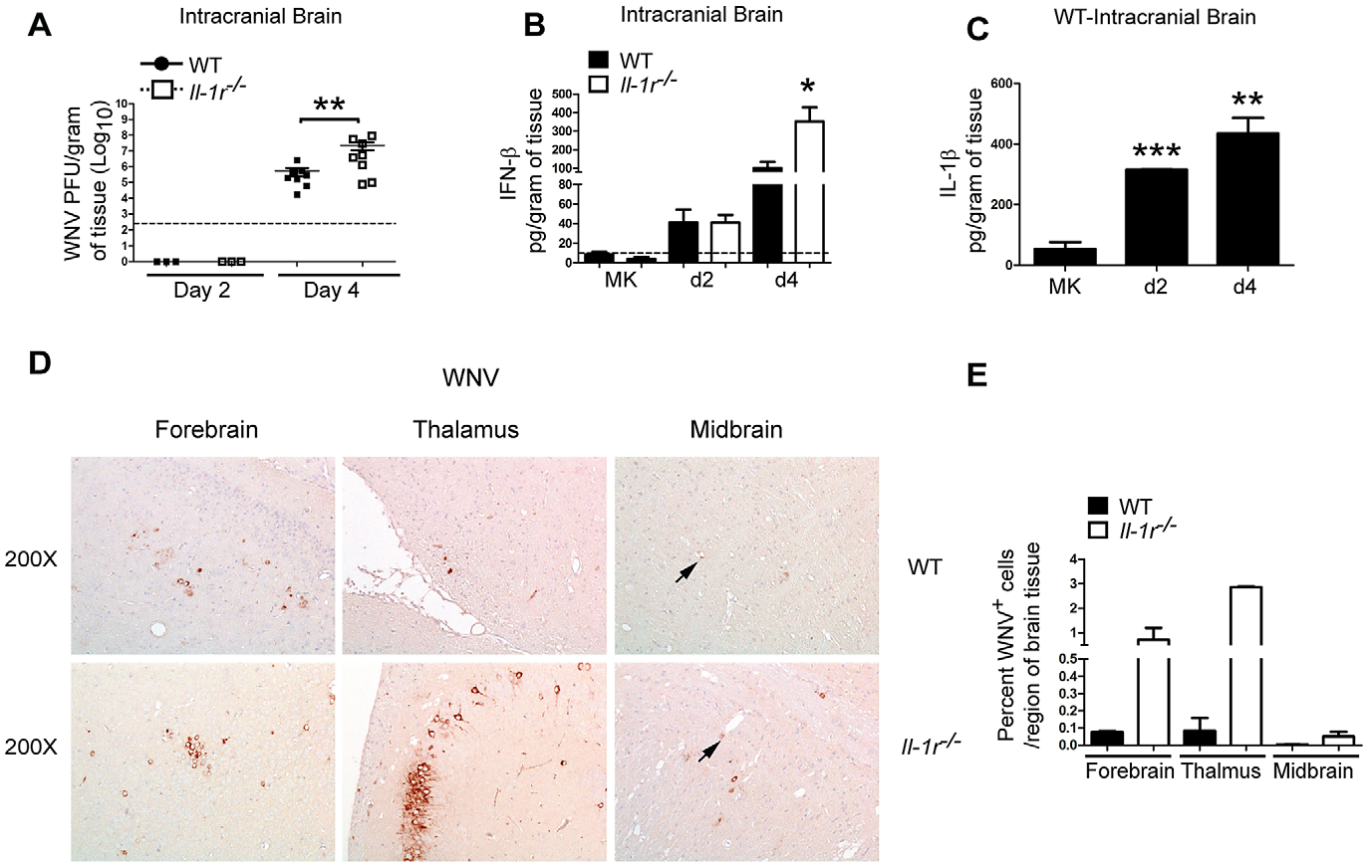
IL-1β signaling mediates CNS-intrinsic control of WNV. WT (closed circles) or *Il-1r^−/−^* (open squares) animals were infected with 5 pfu WNV-TX via direct intracranial (i.c.) inoculation and were examined for viral load and cytokine responses (**A–C**). Viral load was assessed at day 2 or day 4 p.i. in brain (**A**) homogenates by plaque assay. IFN-β secretion was quantified from whole brain lysate from mock or infected at day 2 and day 4 p.i. by ELISA (**B**). Quantification of IL-1β expression from mock or i.c. infected brains of WT animals was assessed at day 2 and day 4 p.i. by Luminex array (**C**). Data are shown as Mean+/− S.E.M. and is displayed as n = 3–7 mice per time-point from two independent pooled experiments (**A**) or n = 3–4 mice (**B,C**) per time-point, representative or three independent experiments. *p<0.05, ** p<0.005, *** p<0.0005. Dashed line represents the lower limit of detection for each assay. Immunohistochemical analysis day 4 i.c. sagittal cut brain sections from paraffin embedded tissue. Representative WNV-Ag (brown staining; **D**) in regions as indicated in WT (top panels) or *Il-1r^−/−^* brain sections (bottom panels). (**E**) Quantification of panel D.

To determine whether IL-1β signaling acted directly within the CNS to control WNV replication, mice were challenged with a low dose of WNV-TX (5 PFU), via intracranial (i.c.) inoculation and assessed for viral load and cytokine responses within the CNS. Similar to our observations in peripheral challenge, *Il-1r^−/−^* mice showed a significant enhancement of viral load in the brain (Figure 7A) as well as spinal cord (data not shown) when compared to WT, i.c. infected, animals, suggesting that IL-1β signaling imparts CNS-intrinsic control of WNV replication. In agreement with this notion, brain tissue sections stained for WNV-antigen showed more widespread and increased intensity staining in *Il-1r^−/−^* when compared to those from WT animals (Figure 7D,E). Staining of viral antigen was associated primarily in cortical, hippocampal and mid-brain neurons with sparse staining in the endothelium (Figure 7D; black arrows indicate staining in endothelial cells) demonstrating a distinct tropism for neuronal cells. Similar to peripherally challenged mice (Figure 3F) the expression of IFN-β was enhanced at day 4 p.i in *Il-1r^−/−^* animals (Figure 7B). Furthermore, in WT mice, i.c. WNV infection triggered IL-1β production in the brain by day 2 p.i. and this was maintained through day 4 p.i. (Figure 7C). Thus, these data indicate that CNS innate immune signaling and IL-1β production are triggered directly by WNV infection and reveal that IL-1β signaling is an essential component of CNS-intrinsic virus restriction.

### IL-1β signaling controls West Nile virus infection in cortical neurons

To determine how IL-1β signaling mediates CNS-intrinsic control of WNV, we examined WNV infection *ex vivo* in cortical neurons, a primary target cell for WNV replication within the CNS. While a direct antiviral role for IL-1β signaling in neurons has not yet been defined, a previous study demonstrated that neurons from *Myd88* ^−/−^ animals presented with increased viral load after WNV infection when compared to their WT counterparts [19]. Thus, we reasoned that Myd88-dependent IL-1β signaling might promote an antiviral state in these cells. Cortical neurons were prepared from WT and *Il-1r^−/−^* mice as previously described [45] and were purified to greater than 95% based on staining for the neuronal marker, NeuN (data not shown). Cultures were infected with WNV (MOI = 1) and single-step growth curve analysis and their host response to WNV infection was examined.

**Figure 8:**
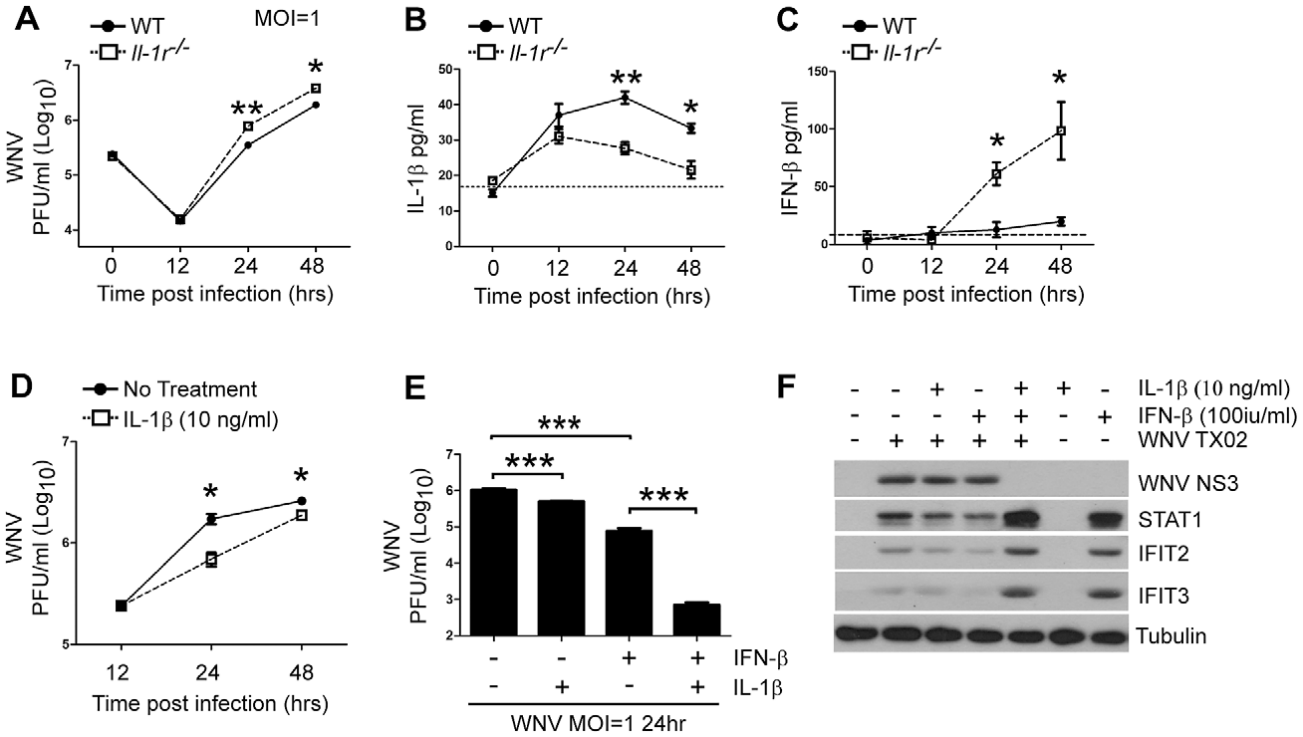
IL-1β synergizes with type I IFN to mediate direct antiviral actions and control of WNV infection in cortical neurons. Cortical neurons were prepared from d15 embryos of WT (closed circles) or *Il-1r^−/−^* (open squares) animals and were assessed for viral load by plaque assay after infection with MOI (1) WNV-TX at 12, 24 and 48 hrs p.i. (**A**). Quantification of IL-1β (**B**) or IFN-β (**C**) from supernatants of mock or WNV MOI 1 infected WT and *Il-1r^−/−^* neurons at 12, 24 and 48 hrs p.i. was assessed by Luminex array (**B**) or ELISA (**C**). IL-1β treatment reduces WNV load in cortical neurons. WT cortical neurons were either infected with MOI1 WNV alone (WT no treatment; closed circles) or with WNV MOI1+24 hr pre-treatment of 10 ng/ml recombinant IL-1β (WT 10 ng/ml IL-1β; open squares), (**D,E**) or with IFN-β (100 IU/ml) or both cytokines together (**E**) and viral load was assessed by plaque assay (**D,E**). Western blot analysis for WNV and interferon stimulated genes with IL-1β and IFN-β treatment (**F**). Data are shown as Mean+/−S.E.M. (**A–E**) for n = 3 per time-point and are representative of three independent experiments. * p<0.05, ** p<0.005, *** p<0.0005. Dashed line represents the lower limit of detection for each assay.

*Il-1r^−/−^* cells showed significantly higher viral loads at 24 hr and 48 hr p.i. when compared to their WT controls (Figure 8A). This increase was also observed in multi-step growth curve analysis after infection with a lower MOI (0.01), although this was not maintained through 48 hrs (Figure S6A). We found that WT neurons produced IL-1β as early as 12 hr p.i. after virus challenge and this persisted throughout the course of infection (Figure 8B), thus suggesting that neurons might contribute in part to the total IL-1β response in the CNS. The production of IL-1β was detectable in *Il-1r^−/−^* cells but at generally lower levels than WT (Figure 8B). Remarkably, we observed enhanced IFN-β production in *Il-1r^−/−^* neurons at both MOIs (Figures 8C, S6B), however this only effective at reducing viral load after low MOI infection (Figure 8A, S6A). These observations demonstrate that IL-1β signaling contributes to the control of WNV infection in cortical neurons and may operate to enhance the antiviral response mediated by type I IFN signaling.

### IL-1β signaling synergizes with type I IFN to control WNV infection in cortical neurons

To determine whether IL-1β could impart suppression of WNV replication in cortical neurons, cells were pre-treated for 24 hrs with either media alone, or IL-1β (10 ng/ml), infected with WNV (MOI = 1) and then assessed for viral load in the supernatant. IL-1β treatment of cells resulted in a reduction of detectable WNV at 24 hr (2.5 fold) and 48 hr (1.7 fold) p.i. compared to non-treated cells (Figure 8D). This level of reduction was similar to the fold increase in viral titers observed in *Il-1r^−/−^* neurons (Figure 8A; 24 hr, 2.3 fold, 48 hr, 2.1 fold) thus demonstrating that IL-1β imparts a response that contributes to the control of WNV replication. In WNV-infected *Il-1r^−/−^* mice we consistently observed increased levels of type I IFN, either in whole tissue (Figures 3F, 7B) or from cortical neurons (Figure 8C, S6B) but this level was insufficient to control the viral load. Thus the actions of IL-1β might function to enhance the antiviral effect of type I IFN against WNV infection.

To test this idea, we conducted experiments in which cortical neurons were either left untreated or pretreated for 24 hrs with IL-1β (10 ng/ml), IFN-β (100 IU/ml) or both cytokines together, followed by WNV infection and assessment of viral load. Similar to our previous results, IL-1β treatment led to a 2.1-fold reduction in virus at 24 hr p.i while treatment of neurons with IFN-β reduced viral load by 13.5-fold at 24 hr p.i., consistent with previous studies [11]–[12] (Figure 8E). Remarkably, when neurons were pretreated with both IL-1β and IFN-β, we observed near complete control of WNV at 24 hr (1500-fold reduction of viral load compared to control; Figure 8E). Further, these results were prolonged as the fold reductions by each cytokine were maintained through 48 hrs p.i. (Figure S6C). We also observed that pre-treatment of neurons with both IL-1β and IFN-β in the context of WNV infection led to an increase in mRNA (Figure S6D) and protein expression (Figure 8F) of IFN-β and interferon stimulated genes (ISGs), STAT1, IFIT1, IFIT2 and IFIT3, molecules known to participate in control of WNV [46]–[47]. This response was comparable to induction by type I IFN in the absence of infection (Figure 8F), suggesting that despite the known ability of WNV to antagonize these responses [44], [48], the synergy of IL-1β and type I IFN might act to overcome this antagonism and promote increased viral control. The induction of ISGs was not observed with IL-1β treatment alone (Figure 8F), demonstrating that combined signals induced by viral infection, type I IFN and IL-1β were required for the synergistic activation of these antiviral genes. Therefore we conclude that IL-1β synergizes with type I IFN to enhance antiviral gene programs that control WNV infection in cortical neurons and thus, IL-1β acts as a key host restriction factor in the control of WNV infection.

## Discussion

Our observations support a model in which IL-1β signaling functions as a host restriction factor to control WNV replication within the CNS. WNV restriction by IL-1β occurs in a manner dependent on the ability of IL-1β to synergize with type I IFN to promote a robust antiviral program in neurons. Further, the capability for IL-1β signaling to control viral load is essential for regulating protective CNS inflammation to control WNV disease, as a lack of IL-1β signaling associates with a breakdown of immunity marked by rapid and uncontrolled viral spread through the CNS, hyper-active inflammatory response and defective CD8^+^ T lymphocyte effector activity. Thus, IL-1β is fundamental for the control of WNV infection and immunity.

To date, three distinct inflammasomes have been described to participate in IL-1β activation in response to viral infection. These include the NLRP3 [32], [34], [36], [49], RIG-I [50] and AIM2 [51]–[53] inflammasomes. While AIM2 is responsible for activation to DNA viruses, both NLRP3 and RIG-I have been associated with IL-1β activation in response to RNA viruses (reviewed in [24]). We found that the defects in immunity observed in *Il-1r^−/−^* mice infected with WNV were pheno-copied in mice deficient in NLRP3 or Caspase-1 suggesting that the NLRP3-inflammasome acts as the predominant pathway for triggering IL-1β production *in vivo* during WNV infection. This is not surprising as NLRP3 activation of IL-1β has been shown to occur in response to multiple RNA and DNA viruses including influenza, Sendai and adenoviruses [24] as well as JEV, a WNV related *Flavivirus* [41]. Further, our observations that NLRP3 signaling is important for limiting lethality and tissue destruction during WNV infection *in vivo* is consistent with models of influenza infection which have also shown a requirement for NLRP3 and IL-1β signaling in protective immunity against the virus [32], [34], [36] as well as in limiting collagen deposition and necrosis of lung tissue [34] and thus demonstrate a broad range for NLRP3 signaling in immunity to virus infection. While NLRP3 and Caspase-1 deficient animals succumbed to WNV infection with similar kinetics and frequency we did observe a trend for increased virulence when compared to *Il-1r^−/−^* animals (Figure 2A,D). While this maps the majority of IL-1 signaling to NLRP3 activation, it is possible that other pathways might contribute to this response. As RIG-I has recently been shown to mediate both the activation of “signal 1” as well as “signal 2” in response to vesicular stomatitis virus (VSV) in an NLRP3-independent manner [50] and RIG-I [9], [44], [54] plays important roles in sensing and triggering of innate immune pathways against WNV virus, it is possible that this pathway may contribute in addition to NLRP3 in the full activation of IL-1β activation during WNV infection *in vivo*.

The contribution of IL-1 signaling to protective immunity against virus infections has largely been attributed to its ability to drive chemokine signaling pathways that recruit immune cells to sites of viral replication [38], [39], [40]. Although CNS recruitment of CD45^+^ monocytes and T lymphocytes was largely increased during WNV infection, in the absence of IL-1 signaling, we did observe a reduced frequency and total number of these cells at day 7 post infection (Figure 4) and this is consistent with previous studies that showed that IL-1β was important for optimal cellular recruitment to the DLN after WNV infection [8]. Therefore, it is possible that this reduction in initial leukocyte and lymphocyte recruitment is sufficient to allow for enough virus to seed infection in neurons and increase the likelihood of CNS spread. Interestingly, despite early differences in cellular recruitment and inflammation in the CNS, by later time-points, we observed a dramatic enhancement in inflammation in *Il-1r^−/−^* animals (Figure 4). This is in contrast to recent studies examining WNV infection in Myd88 [19] and TLR7 [20] deficient animals which show dramatic reduction in cellular infiltrates and inflammation within the CNS. Thus, it is likely, that while IL-1β signaling contributes to cellular recruitment, TLR signaling via MyD88 might play a dominant role in maintaining these responses over time.

As CD8^+^ T cells represent a key component in late CNS control of WNV, it is interesting that IL-1 signaling defects were associated with decreased effector activity of these cells. Our data suggest that increased viral load influence this defect directly by driving increased inflammatory cytokine responses and a reduced frequency of antigen-specific cells to total CD8^+^ cells. However, in addition, it is also possible that IL-1β signaling itself is required to directly promote the optimal T cell effector response. This outcome is similar to observations of influenza infection in which IL-1R and NLRP3-inflammasome deficient animals display defective CD4^+^ and CD8^+^ T lymphocyte responses in the absence of increased inflammation [36]. Furthermore, MyD88 signaling was found to act in a T cell intrinsic manner to control both proliferation and acquisition of effector responses during LCMV [55]–[56] and vaccinia virus infection [57]–[58], while direct IL-1 signaling in has been shown to influence the development of multiple T lymphocyte effector populations under multiple experimental conditions [23]. Our data are in agreement with a role for IL-1β signaling in driving T cell effector activity during viral infection. Such action might occur against WNV infection by either of two mechanisms: First, IL-1β signaling could indirectly limit inflammation and overstimulation of cells in the CNS that otherwise occurs under conditions of uncontrolled virus replication and dissemination [9], [19]–[20]. Secondly, IL-1β signaling could directly drive a response, likely in a Myd88-dependent manner, to positively regulate immune cell effector activity against WNV.

We observed a significant reduction in type I IFN in the DLN at day 2 p.i. in *Il-1r^−/−^* mice that correlated with a modest increase in viral load (Figures S3D, 3A). This result is notable because the observation occurred at a time-point that is associated with the accumulation of macrophages and DC subsets trafficking into the DLN from the site of primary infection, wherein type I IFN signaling is associated with control of the virus in these cell types [9]. We have also observed reduced type I IFN responses and increased viral replication in primary *Il-1r^−/−^* macrophages and DCs infected with WNV *in vitro* (Ramos, HJ; unpublished observations). Furthermore, it has been proposed that viral entry to the CNS could in part be mediated by a “Trojan horse” mechanism in which immune cells harboring WNV infiltrate into the CNS and seed viral infection in neurons [5]. Together, these observations raise the possibility that the lack of type I IFN in the DLN in *Il-1r^−/−^* mice is associated with decreased control of virus within macrophages and DCs, which then infiltrate and seed the CNS with virus in an enhanced manner that promotes the robust inflammatory disease observed in *Il-1r^−/−^* mice. In this sense IL-1β would serve to control virus levels in infected inflammatory cells and thereby restrict virus seeding into the CNS that might occur through infiltration of infected cells.

Type I IFN and to a lesser extent, IFN-γ, and TNF-α, have also been shown to mediate anti-viral responses to WNV [13], [18] and our results now implicate IL-1β as a component of innate antiviral signaling and response against WNV infection. In support of this, a protective role for IL-1β in combination with TNF-α was shown to be important to the control of hepatitis B virus [21]–[22]. In addition, *in vitro* studies in hepatocyte cell lines revealed that this effect was mediated by the ability of IL-1β to augment type I and type II IFNs antiviral activities in mechanisms dependent on STAT-1 and P38 MAP kinase [21]. This phenomenon of IL-1β synergy has also been observed in epithelial cell infection with VSV [37]. Therefore, we propose that in the context of WNV infection, IL-1 acts synergistically with type I IFN, and possibly type II IFN and/or TNF-α to mediate an antiviral program to control WNV in the CNS.

We found that IL-1β treatment of WT cortical neurons *ex vivo* resulted in reduced levels of detectable virus in the supernatants of infected neurons however, this response was not sufficient by itself to fully inhibit the virus. Interestingly, the viral suppression we observed occurred between 24 and 48 hrs of treatment suggesting that IL-1β signals a response in neurons that restricts virus amplification rather than initial infection. This kinetics are suggestive of a mechanism by which products of IL-1β -responsive genes serve to impart control of WNV replication, thus suppressing virus spread within the CNS. Indeed, our data demonstrate that IL-1β acts synergistically in the innate antiviral response along with IFNs and possibly other cytokines to ultimately control virus replication. In line with this notion, we observed that IL-1β and type I IFN synergized to enhance the expression of ISGs such as the IFIT family members IFIT1,2,3. This is of interest as recent studies have implicated these antiviral effectors in the control of CNS specific viruses such as VSV [59]. Furthermore, recent data had identified IFITs as important in anti-viral control of WNV [46]–[47] and this is dependent on their ability to block the viral replication cycle [46]. Therefore, IL-1β might control WNV through its ability to modulate ISGs that impart antiviral actions against WNV replication.

Control of WNV replication in the CNS is paramount for protection against disease. Our study shows that IL-1β production and signaling are important for protective immunity against WNV suggesting that the NLRP3-inflammasome and IL-1 signaling also likely impact immunity to other *Flaviviruses*. It has been observed that dengue, yellow fever (YFV), St. Louis encephalitis (SLEV) and WNV all trigger IL-1β expression from populations of myeloid derived cells *in vitro*, while YFV, SLEV and WNV have each been linked to suppression of IL-1β signaling at various levels *in vitro* [60]–[62]. Taken together, these observations along with those made in this study identify IL-1β as a key host restriction factor in immunity against *Flavivirus* infection.

## Methods

### Ethics statement

All animal experiments were approved by the University of Washington Institutional Animal Care and Use Committee (IACUC) committee guidelines (protocol number: 4158-01) and follow the recommendations in the Guide for the Care and Use of Laboratory Animals of the National Institutes of Health. Mouse infections and manipulations were performed under anesthesia of ketamine and xylazine, and every effort was made to limit suffering.

All human subjects provided written-informed consent under a University of California, San Francisco Institutional Review Board approved protocol.

### Viruses and cell lines

WNV isolate, TX 2002-HC (WNV-TX02), was titered by a standard plaque assay on BHK-21 cells and working stocks of WNV-TX were generated as previously described [9]. BHK-21 cells were cultured in Dulbecco's modified Eagle medium (DMEM) supplemented with 10% fetal bovine serum (FBS), HEPES, L-glutamine, sodium pyruvate, antibiotic-antimycotic solution, and nonessential amino acids.

### Human subjects

The 43 WNV^+^ subjects included in this study were enrolled in 2009 and 2010 by Blood Systems Research Institute (BSRI) through the United Blood Services network of blood centers. Blood donors who tested positive for WNV RNA by routine donation screening using the WNV Procleix Transcription Mediated Amplification (TMA) assay were asked to return to their local blood donation center for enrollment after informed consent was completed under a University of California, San Francisco Institutional Review Board approved protocol. Upon enrollment, blood donors agreed to return to their blood center for subsequent follow up visits. Infection was confirmed using follow-up samples showing sero-conversion to anti-WNV IgM. Samples were collected at regional blood centers and were shipped by overnight courier to BSRI. Blood was processed within 24 hours and plasma aliquots were frozen immediately for long term storage. The WNV^+^ subjects were 58% male with an average age of 51 years old. The control subjects (Cntrl.) used in this study were 21 BSRI staff members who consented to donate blood for this study and they were 43% male with an average age of 48 years old.

### WNV viral load quantification

Quantification of WNV viral load in follow-up human plasma specimens was assayed by real-time reverse transcription-polymerase chain reaction as previously described [42].

### *In vivo* murine infections

C57BL/6 (WT) and IL-1Rα deficient mice were purchased from Jackson Laboratories, Bar Harbor, ME. NLRP3 [63], Caspase-1 [64] and NLRC4 [65] were generously provided by Dr. Vishva Dixit (Genentech, San Francisco, CA), Dr. Chris Wilson (University of Washington, Seattle, WA) and Dr. Alan Aderem (Seattle Biomed, Seattle, WA). All mice were genotyped for positive identification and were bred in specific pathogen-free conditions in the animal facility at the University of Washington. Experiments were performed in accordance with the University of Washington Institutional Animal Care and Use Committee guidelines. Age-matched six to ten week old mice were inoculated subcutaneously (s.c.) in the rear footpad with 100 PFU of WNV-TX 02 in a 10 µl inoculum diluted in phosphate buffered saline (PBS) supplemented with 1% heat-inactivated FBS. Mice were monitored daily for morbidity and mortality. For clinical scoring, infected mice were monitored daily for signs of hind limb dysfunction and paresis. Mice were scored using the following scale from 1–6: 1, ruffled fur/lethargic, no paresis; 2, very mild to mild paresis; 3, frank paresis involving at least one hind limb and/or conjunctivitis; 4, severe paresis; 5, true paresis; 6, moribund.

### Viral tissue quantification

To determine *in vivo* viral burden, s.c infected mice were euthanized, and perfused with 20–30 ml of PBS to remove blood from tissues. Whole tissue were removed, weighed, and homogenized in 500 µl (spleen, brain) or 200 µl (spinal cord) of PBS containing 1% heat-inactivated FBS using a Precellys 24 at 1500 RPM for 20 seconds (Bertin Technologies, France). Sample homogenates were then titered by plaque assay on BHK cells as previously described [9]. For analysis of viral load within the draining lymph nodes (DLN), the popliteal DLN was harvested and homogenized as described above in 350 µl of RNA extraction buffer (RLT, Qiagen), and total RNA was extracted using an RNeasy kit (Qiagen). DNase treated RNA (Qiagen) was then reversed transcribed to cDNA using a 1∶1 mixture of random hexamers and oligodT primers with the iScript select cDNA synthesis kit (Biorad). WNV-specific RNA copy number was measured by single-step Real Time-quantitative PCR (qRT-PCR) using Taqman technology via specific primer sets and probes as previously described [9]. For serum samples, viral RNA was isolated from 50 µl of serum from mock or WNV-TX infected samples using the QIamp Viral RNA isolation kit (Qiagen). Isolated viral RNA was then subjected to cDNA synthesis and qRT-PCR for assessment of WNV copy number as described above.

#### Primary cell isolation and infection

Cortical neurons were isolated from 15-day-old embryonic mice and cultured as described previously [45] in Neurobasal media (Invitrogen) supplemented with B27 supplement (Invitrogen), HEPES, L-glutamine and antibiotic-antimycotic solution and plated on Laminin coated plates. On day 4 of culture, neurons were infected with WNV-TX02 at an MOI of 0.01 or 1 and supernatants were collected for evaluating viral titers. Cells were collected for RNA analysis by RT-qPCR using specific primer sets. In addition, cell pellets were lysed in 30 µl radioimmunoprecipitation assay (RIPA) buffer for use in western blot analysis. For anti-viral assays, neurons were treated on day 4 post isolation for 24 hrs with either IL-1β alone, 10 ng/ml (Miltenyi biotec), IFN-β, 100 IU/ml (PBL interferon source), both cytokines or left untreated. After 24 hrs, supernatant was removed and cells were infected as described above.

### Protein analysis

Whole brain tissue was isolated from mice perfused as described above. Tissue was lysed in 1 ml per brain RIPA buffer containing a cocktail of protease and phosphatase inhibitors (Sigma). Lysis was facilitated by homogenization using the Precellys (Bertin Technologies, France) as described above. Protein extracts (20 µg) were analyzed by immunoblotting. The following primary antibodies were used to probe blots: goat anti-WNV NS3 (R&D systems); rabbit anti-ISG54 (IFIT2) and rabbit anti-ISG49 (IFIT3), kindly provided by Dr. G. Sen; mouse anti-tubulin (Sigma) and rabbit anti-STAT1, (Cell Signaling). Secondary antibodies included peroxidase-conjugated goat anti-rabbit, donkey anti-goat and goat anti-mouse (Jackson Immunoresearch).

### RNA extraction and analysis

Total RNA was extracted from tissues and cDNA was generated as described above. Cytokine expression was then assessed by one-step SYBR Green RT-qPCR using an ABI 7800 machine. Similarly RNA was extracted from neurons using 350 µl/sample buffer RLT as described for DLN samples. Specific primer sets for GAPDH, IL-1α, IL-1β, IFNβ, TNFα, and IL-6 are described as follows:

**mGAPDH forward**: CAACTACATGGTCTACATGTTC, **mGAPDH** reverse: CTCGCTCCTGGAAGATG; **mIFNβ** forward: GGAGATGACGGAGAAGATGC **mIFNβ** reverse: CCCAGTGCTGGAGAAATTGT; **mIL1α forward**:TCTATGATGCAAGCTATGGCT, **mIL-1α** reverse: CGGCTCTCCTTGAAGGTGA; **mIL1β** forward: ACGGACCCCAAAAGATGAAG, **mIL1β** forward: CACGGGAAAGACACAGGTAG; **mIL6 forward**: GTTCTCTGGGAAATCGTGGA, **mIL6 reverse**: TGTACTCCAGGTAGCTATGG; **mTNFα forward**: CATCTTCTCAAAATTCGAGTGACAA, **mTNFα reverse**:TGGGAGTAGACAAGGTACAACCC; mIFIT1, mIFIT2 and mIFIT3 were purchases as pre-mixed SuperArray primer sets (Qiagen).

#### IFN-β ELISA

For detection of IFN-β in cell culture supernatants, 100 µl of UV-inactivated supernatant was tested using mouse-specific ELISA kits from PBL Biomedical Laboratories according to the manufacturer's protocol.

### WNV-specific antibody ELISA and PRNT analysis

WNV-specific IgM and IgG, levels were determined by an ELISA using purified recombinant E protein as previously described [66]. The neutralization titer of serum antibody was determined using the plaque reduction neutralization assay as previously described [67]. The dilution at which 50% of plaques were neutralized was determined by comparing the number of plaques formed from WNV-infected sera samples to mock infected sera samples.

### Histological analysis

Mock-infected or WNV-infected mice were sacrificed by exsanguination and perfused with PBS-4% paraformaldehyde, pH 7.3. Brains were embedded in paraffin and 4–6 µm sections were prepared and stained with hematoxylin and eosin (H&E) or immunohistochemistry by the UW Histology and Imaging Core. H&E-stained sections were evaluated for viral-induced neuropathology. Antibodies for immunohistochemical detection included for for macrophages, rat anti mouse MAC-2, Clone M3/38, (Cedarlane, Cat No. CL8942AP); and for West Nile virus, rabbit anti-WNV, clone 7H2 + rabbit anti-WNV polyclonal (BioReliance, Cat No. 81-002, 81-015). Immunohistochemical staining was performed on a Leica Bond Automated Immunostainer with Leica Refine (DAB) detection and hematoxylin counterstain. Images were captured using a Nikon 80i Eclipse microscope with a digital camera with NIS Elements Basic research imaging software. Quantitation for MAC-2 and WNV was performed with the Visiopharm histoinformatics software (Visiopharm) on representative regions of two sets of brain sections and is representative of the ratio of specific staining to the total area of the tissue..

For quantification of WNV and MAC-2, slides were scanned in Brightfield at a 20× objective using the Nanozoomer Digital Pathology slide scanner (Hamamatsu; Bridgewater, New Jersey). The digital images were then imported into Visiopharm software (Hoersholm, Denmark) for analysis. Using the Visiomorph Digital Pathology module, regions of interest (ROIs) were applied around the relevant areas on each slide and consisted of one rectangular ROI within each of three areas—the forebrain, midbrain, and thalamus—on each section. The images were processed in batch using these configurations to generate the desired outputs (areas of staining and normal tissue, in square microns), from which the percent of WNV or MAC2 staining was calculated.

### Tissue isolation and flow cytometric analysis

Splenocytes were isolated by grinding on frosted glass slides, washed counted, and re-suspended in RPMI 1640 containing 10% FBS before cell surface staining or *in vitro* stimulation. For staining, cells were washed once in PBS and once in PBS+0.5% BSA (FACS wash) in a 96 well plate format and then stained in 50 µl FACs wash plus directly-conjugated antibodies specific to CD8, CD4, and CD3 (Biolegend). WNV-specific CD8^+^ T cells were identified using a Db-restricted NS4b peptide tetramer directly conjugated to either Phycoerythrin (PE) or allophycocyanin (APC). For intracellular cytokine staining, cells were stimulated with 1 µM of the immune-dominant peptide NS4b (SSVWNATTA) for 4 hrs at 37°C in the presence of golgi-stop (BD-PharMingen). After stimulation, cells were washed twice in FACS wash and stained for cell surface markers followed by permeabilization-fixation using the Cytofix-Cytoperm Kit (BD-PharMingen) and stained with a Pacific Blue-conjugated IFN-γ and FITC-conjugated TNF-α antibody (eBiosciences) or FITC-conjugated perforin and PE-CY7-conjugated granzyme B at 4°C for 30 min, washed and analyzed by flow cytometry. Flow cytometry was performed on a BD LSRII machine using BD FACSDiva software. Cell analysis was performed on FlowJo (v.8.7.2) software.

For isolation of CNS immune cells, euthanized mice were perfused with 20–30 ml of PBS to remove residual intravascular leukocytes. Brains were isolated and minced in RPMI media, rigorously triturated, and digested with Liberase (Roche) and type I DNase in serum-free RPMI media at 37°C for 1 hr. Immune cells were isolated after gradient centrifugation over 25% Percoll followed by a wash and secondary centrifugation over a 30% Percoll gradient. Cell pellets were isolated and washed twice and counted. Cells were resuspended in 50 µl FACS wash and stained for surface expression of CD4, CD8, CD33, CD11b and CD45 (Biolegend). For intracellular staining, cells were stimulated with 1 µM of NS4b peptide (SSVWNATTA) and stained as described above for IFN-γ, TNF-α, and granzyme B and perforin. Cells were analyzed as described above for splenocytes.

### ELISA and Luminex

Mice were euthanized and blood was collected for serum preparation via exsanguination. Serum was isolated by separating in microtainer (BD Biosciences) at 10,000 rpm for 8 minutes. Brain, spleen, and lymph nodes were isolated and weighed after perfusion with 20 ml of PBS and homogenized using the Precellys (Bertin Technologies, France) as described above in 1 ml of RIPA buffer. Protein concentration was assessed by Bradford colorimetric assay (Biorad) and 200 µg of total protein were loaded for IL-1β (Biolegend), IL-1α (Biolegend) and IFNβ ELISA (PBL Biomedical Laboratories) via the manufactures protocol. Concentration of cytokine was then normalized to the weight of total tissue. For Luminex, 12.5 µl of tissue lysate or serum was run on an 11-plex assay (Miltenyi Biosciences) followed by analysis on a Bioplex 200 (Biorad). Concentration of cytokine was then normalized to total weight of tissue.

For human plasma cytokine analysis, plasma samples were assayed for IL-1β (0.06 pg/ml) and TNF-α (0.05 pg/ml) using the High Sensitivity Human Cytokine kit (Millipore, Billerica, MA, USA), which has a minimum detectable concentration as indicated above and for TNF-α, and for IFN-γ (0.1 pg/ml), IFN-α2 (24.5 pg/ml) IL-1ra (2.9 pg/ml), andIL-1α (3.5 pg/ml) using the Human Cytokine/Chemokine kit (Millipore) which has minimum detectable concentrations as indicated above. Plasma samples were assayed following the manufacturer's protocols. Standard curves were run in duplicate, and samples were tested in duplicate. Acquisition was done on a Labscan 100 analyzer (Luminex) using Bio-Plex manager 6.1 software (Bio-Rad).

### Statistical analysis

For *in vivo* viral burden analysis Kaplan-Meier survival curves were analyzed by the log-rank test. For all *in vitro* studies statistical analysis was performed via unpaired two-tailed student T-test. For *in vivo* viral burden and immune cell analysis experiments statistical significance was calculated using the Mann-Whitney test. A *p*-value≤0.05 was considered significant.

All data were analyzed using Prism software (GraphPad Prism5).

For human patient studies, cytokine levels were compared between WNV^+^ subjects and ^-^ normal controls at each time-point using one-way analysis of variance (ANOVA). Trend analyses were used to evaluate whether a statistically significant increase or decrease of cytokine levels was observed in the time post-index within the WNV^+^ subjects; to detect monotonously increasing or decreasing trend of cytokine quantities over time, Page's trend tests were applied on each cytokine using the R Package ‘concord’. A generalized linear model with repeated measures (Proc genmod (GEE) SAS 9.2) was used to analyze in plasma the correlation between WNV viral load and levels of cytokines/chemokines measured at one, three, and six weeks post-index donation. Statistical significance was defined as a *p*-value≤0.05.

## Supporting Information

Figure S1 **Cytokine production during acute West Nile Virus infection in humans.** IL-1 in plasma of WNV infected (n = 43) and control subjects (n = 21). Plasma from blood donors testing positive for WNV RNA were collected at time-points from 7 to 180 days post-Index and was compared to control (cntrl) samples. IFN-γ (**A**), TNF-α (**B**), or IL-1α (**C**) by luminex cytokine bead array. Middle bars represent the median for each group. * p<0.05, ** p<0.005, *** p<0.0005 values are reflective of significance compared to control samples. ns refers to not-significant. Dashed lines represent the lower the minimal detectable concentration of the assay.
(TIF)

Figure S2 **IL-1 signaling and the NLRP3 inflammasome limit CNS disease during WNV infection.** Examination of CNS disease in 6–10 wk old age matched WT (closed circles) or *Caspase-1^−/−^*(**A,B**), *Nlrp3^−/−^*(**C,D**), *Nlrc4^−/−^* (**E,F**) and *Myd88^−/−^* (**G,H**) (open circles) animals. Respective mice were infected with 100 PFU WNV-TX and were monitored daily for weight loss (**A,C,E,G**) or scored for hind limb paralysis and morbidity (**B,D,F,H**) to day 16 post infection.
(TIF)

Figure S3 **Expression of IL-1 and IFN-β in tissues associated with WNV replication.** Examination of *in vivo* cytokine expression and viral load in WT and *Il-1r^−/−^* animals. 6–10 wk old WT mice were infected s.c. with 100 PFU WNV-TX or mock infected and the kinetics of expression of IL-1β (closed circles) or IL-1α (open squares) (**A,B**) (WT only) or IL-1β (**C**) or IFN-β (**D**) in (WT or *Il-1r^−/−^*) mice were assessed. Cytokine expression was assessed by quantitative real-time PCR (qRT-PCR) using specific primers for IL-1α and IL-1β (**A**) or IFN-β (**D**) and made relative to GAPDH and normalized to mock values in the draining lymph node (DLN) or Luminex array for spleen (**B**) and Brain (C). Data are shown as the mean +/− S.E.M. for n = 3–6 mice per time-point. *p<0.05, *** p<0.0005. Dashed lines represent the lower limit of detection for each assay. BLD denotes below limit of detection.
(TIF)

Figure S4 **Deficiency in IL-1β signaling is associated with decreased CNS control of WNV.** Examination of viral loads in the CNS of WT or IL-1 signaling deficient animals. Mice were infected with 100 PFU WNV-TX and viral loads were assessed in the brain by plaque assay for WT (closed circles) and *Myd88^−/−^* (open squares) (**A**) or *Nlrp3^−/−^* (open squares) (**B**) at day 8 and day 10 p.i. Viral loads in the spinal cord at day 10 p.i. (**C**).
(TIF)

Figure S5 **IL-1β signaling is important for control of inflammatory responses in the CNS during WNV infection.** Assessment of inflammatory responses in the CNS of WT (closed squares) or *Il-1r^−/−^* (open squares) mice. The frequency of leukocyte infiltration into the CNS was assessed by flow cytometry at day 6–10 post infection (p.i.) with WNV-TX (**A–C**). Brains from WT (open squares), *Il-1r^−/−^* (closed squares) or Nlrp3^−/−^ (gray squares) were assessed for cytokines and chemokines at day 8 p.i. by Luminex array (**D–F**). * p<.05, **p<0.005, *** p<0.0005.
(TIF)

Figure S6 **IL1β is associated with antiviral activity in the CNS.** Cortical neurons were prepared from d15 embryos of WT (closed circles) or *Il-1r^−/−^* (open squares) animals and were assessed for viral load by plaque assay (**A**) or IFN-β (**B**) expression by ELISA after infection with low MOI (0.01) WNV-TX at 12, 24 and 48 hrs p.i. (C) The fold reduction in viral titer after IL-1β, IFN-β or IL-1β+IFN-β pre-treatment at 24 hr and 48 hr post infection with WNV-TX. ISG expression was assessed by quantitative Real-time PCR (qRT-PCR) using specific primers for IFIT1 (ISG56), IFIT2 (ISG54), IFIT3 (ISG49) and IFN-β made relative to GAPDH and normalized to mock values at 24 hr p.i. (**D**). Data are shown as Mean+/−S.E.M. for n = 3 per time-point and are representative of three independent experiments (**A,B,D**). For panel **C**, data are shown as Mean+/−S.E.M. for n = 9 and are compiled data from three independent experiments. * p<0.05, ** p<0.005, *** p<0.0005. Dashed line represents the lower limit of detection for each assay.
(TIF)

Table S1 **The antibody response to West Nile virus is not altered in IL-1R or inflammasome deficient animals.** Mice were infected with WNV and serum was isolated at days 6 or 8 p.i. from WT and inflammasome deficient animals. Serum IgG and IgM were detected by ELISA for antibody specific to WNV-E protein. For PRNT assay, serum was used to neutralize purified WNV-TX02 in BHK infections. Data is presented as the dilution at which antibody was detected at three standard deviations above mock or for PRNT, the dilution required to neutralize virus by 50%.
(DOCX)
