# Supplementary figures and images for: IL-1β Signaling Promotes CNS-Intrinsic Immune Control of West Nile Virus Infection
Source: PLoS Pathog. 2012 Nov 29;8(11):e1003039. doi: 10.1371/journal.ppat.1003039 (PMC3510243; doi:10.1371/journal.ppat.1003039)

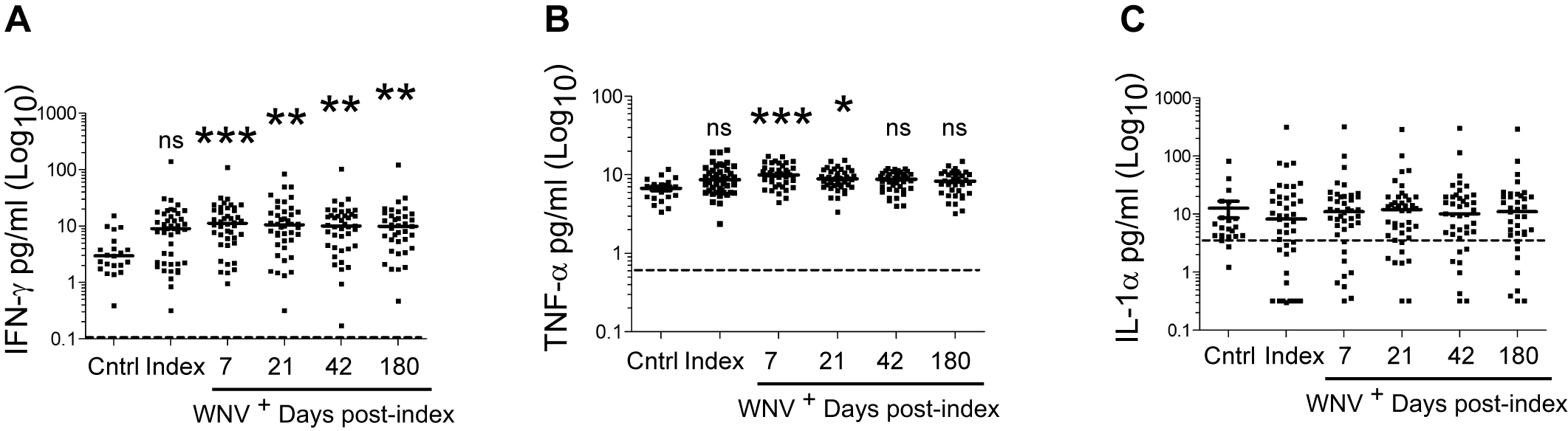

Supplement: Figure S1 — Cytokine production during acute West Nile Virus infection in humans. IL-1 in plasma of WNV infected (n = 43) and control subjects (n = 21). Plasma from blood donors testing positive for WNV RNA were collected at time-points from 7 to 180 days post-Index and was compared to control (cntrl) samples. IFN-γ (A), TNF-α (B), or IL-1α (C) by luminex cytokine bead array. Middle bars represent the median for each group. * p<0.05, ** p<0.005, *** p<0.0005 values are reflective of significance compared to control samples. ns refers to not-significant. Dashed lines represent the lower the minimal detectable concentration of the assay. (TIF) [file ppat.1003039.s001.tif]

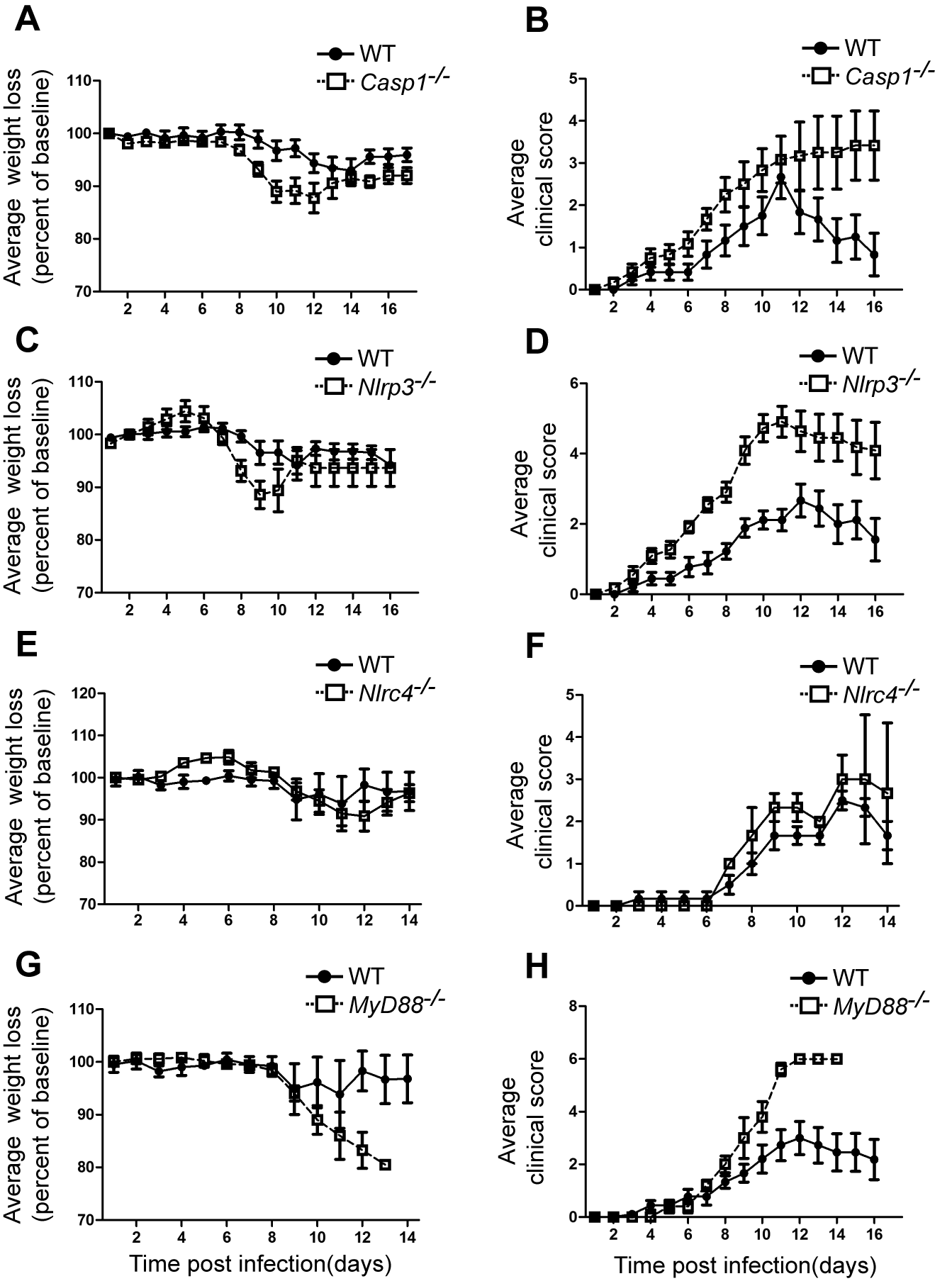

Supplement: Figure S2 — IL-1 signaling and the NLRP3 inflammasome limit CNS disease during WNV infection. Examination of CNS disease in 6–10 wk old age matched WT (closed circles) or Caspase-1−/−(A,B), Nlrp3−/−(C,D), Nlrc4−/− (E,F) and Myd88−/− (G,H) (open circles) animals. Respective mice were infected with 100 PFU WNV-TX and were monitored daily for weight loss (A,C,E,G) or scored for hind limb paralysis and morbidity (B,D,F,H) to day 16 post infection. (TIF) [file ppat.1003039.s002.tif]

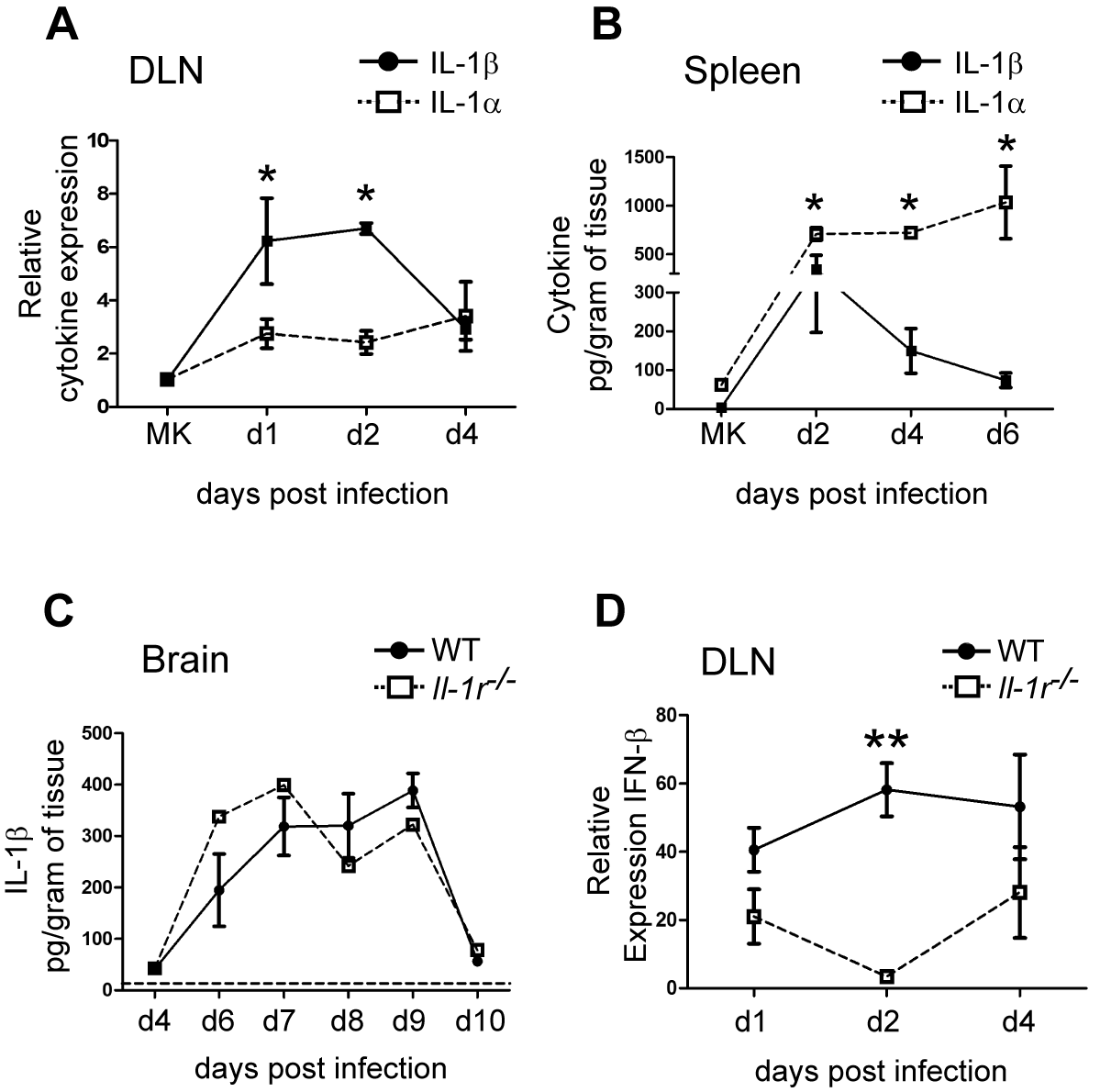

Supplement: Figure S3 — Expression of IL-1 and IFN-β in tissues associated with WNV replication. Examination of in vivo cytokine expression and viral load in WT and Il-1r−/− animals. 6–10 wk old WT mice were infected s.c. with 100 PFU WNV-TX or mock infected and the kinetics of expression of IL-1β (closed circles) or IL-1α (open squares) (A,B) (WT only) or IL-1β (C) or IFN-β (D) in (WT or Il-1r−/−) mice were assessed. Cytokine expression was assessed by quantitative real-time PCR (qRT-PCR) using specific primers for IL-1α and IL-1β (A) or IFN-β (D) and made relative to GAPDH and normalized to mock values in the draining lymph node (DLN) or Luminex array for spleen (B) and Brain (C). Data are shown as the mean +/− S.E.M. for n = 3–6 mice per time-point. *p<0.05, *** p<0.0005. Dashed lines represent the lower limit of detection for each assay. BLD denotes below limit of detection. (TIF) [file ppat.1003039.s003.tif]

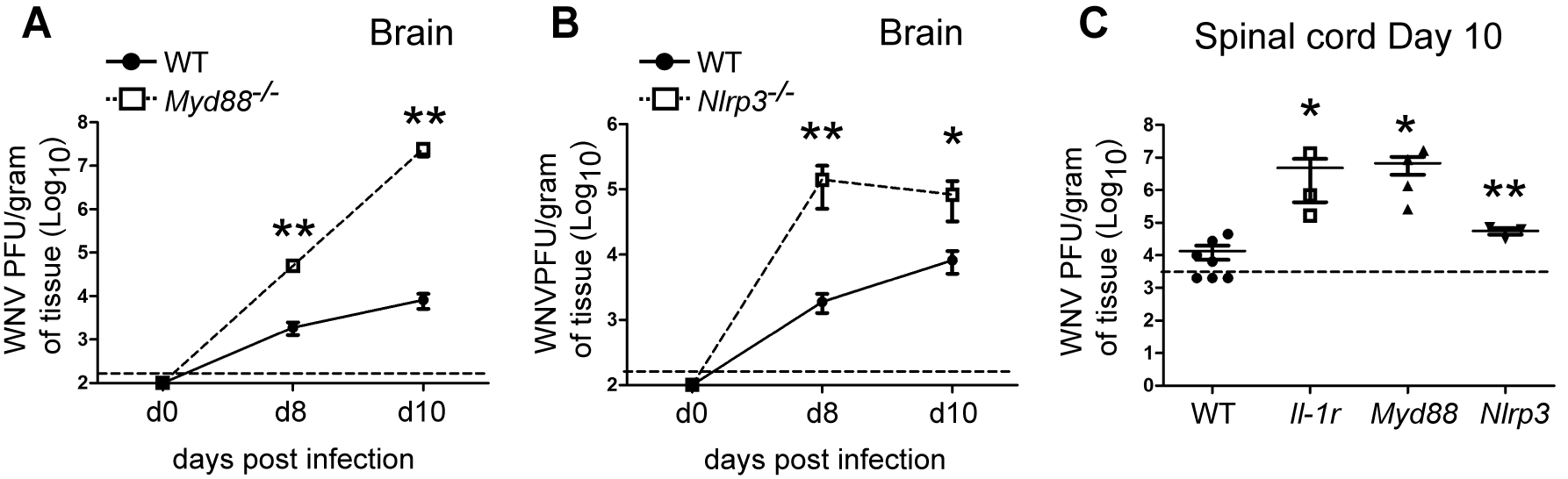

Supplement: Figure S4 — Deficiency in IL-1β signaling is associated with decreased CNS control of WNV. Examination of viral loads in the CNS of WT or IL-1 signaling deficient animals. Mice were infected with 100 PFU WNV-TX and viral loads were assessed in the brain by plaque assay for WT (closed circles) and Myd88−/− (open squares) (A) or Nlrp3−/− (open squares) (B) at day 8 and day 10 p.i. Viral loads in the spinal cord at day 10 p.i. (C). (TIF) [file ppat.1003039.s004.tif]

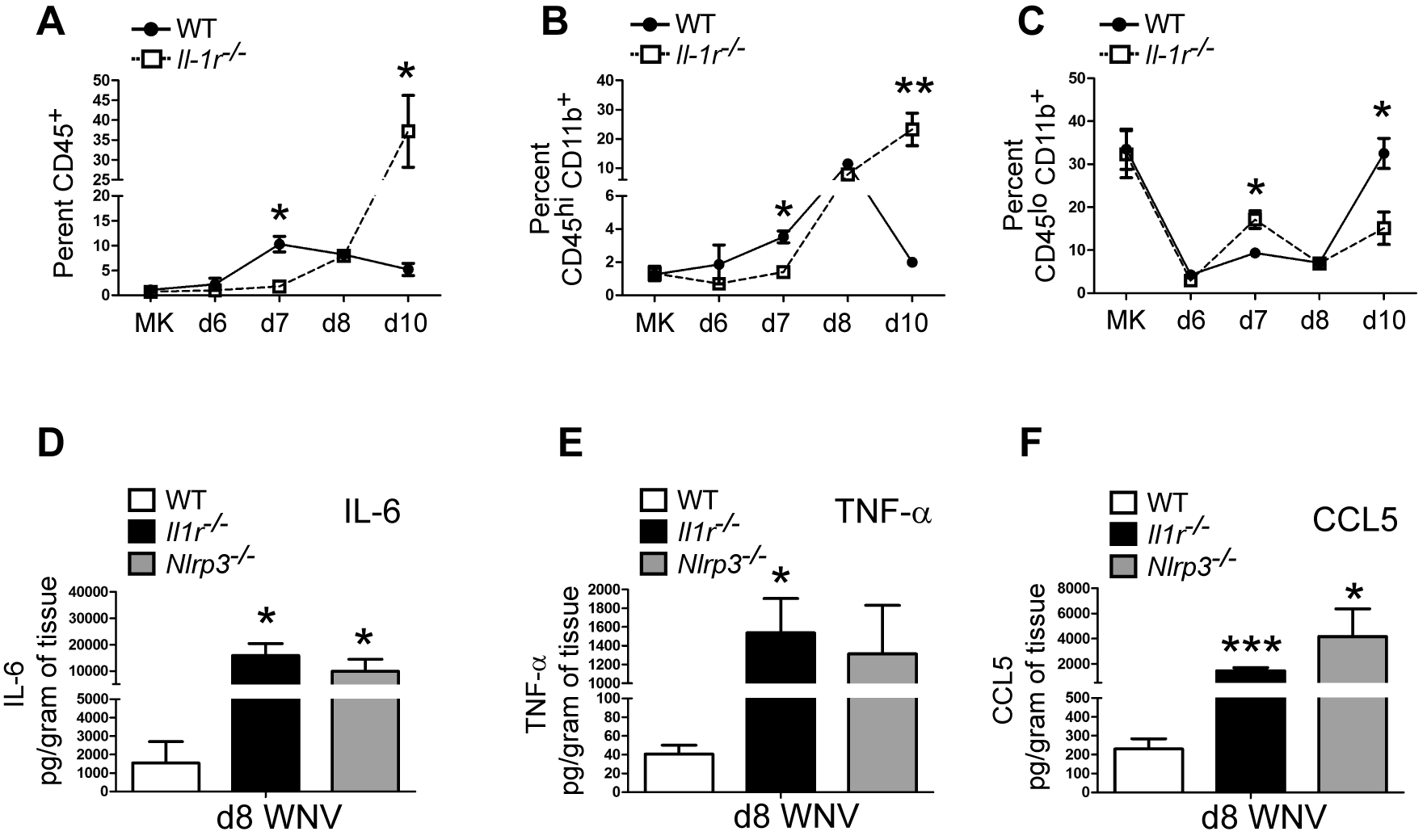

Supplement: Figure S5 — IL-1β signaling is important for control of inflammatory responses in the CNS during WNV infection. Assessment of inflammatory responses in the CNS of WT (closed squares) or Il-1r−/− (open squares) mice. The frequency of leukocyte infiltration into the CNS was assessed by flow cytometry at day 6–10 post infection (p.i.) with WNV-TX (A–C). Brains from WT (open squares), Il-1r−/− (closed squares) or Nlrp3−/− (gray squares) were assessed for cytokines and chemokines at day 8 p.i. by Luminex array (D–F). * p<.05, **p<0.005, *** p<0.0005. (TIF) [file ppat.1003039.s005.tif]

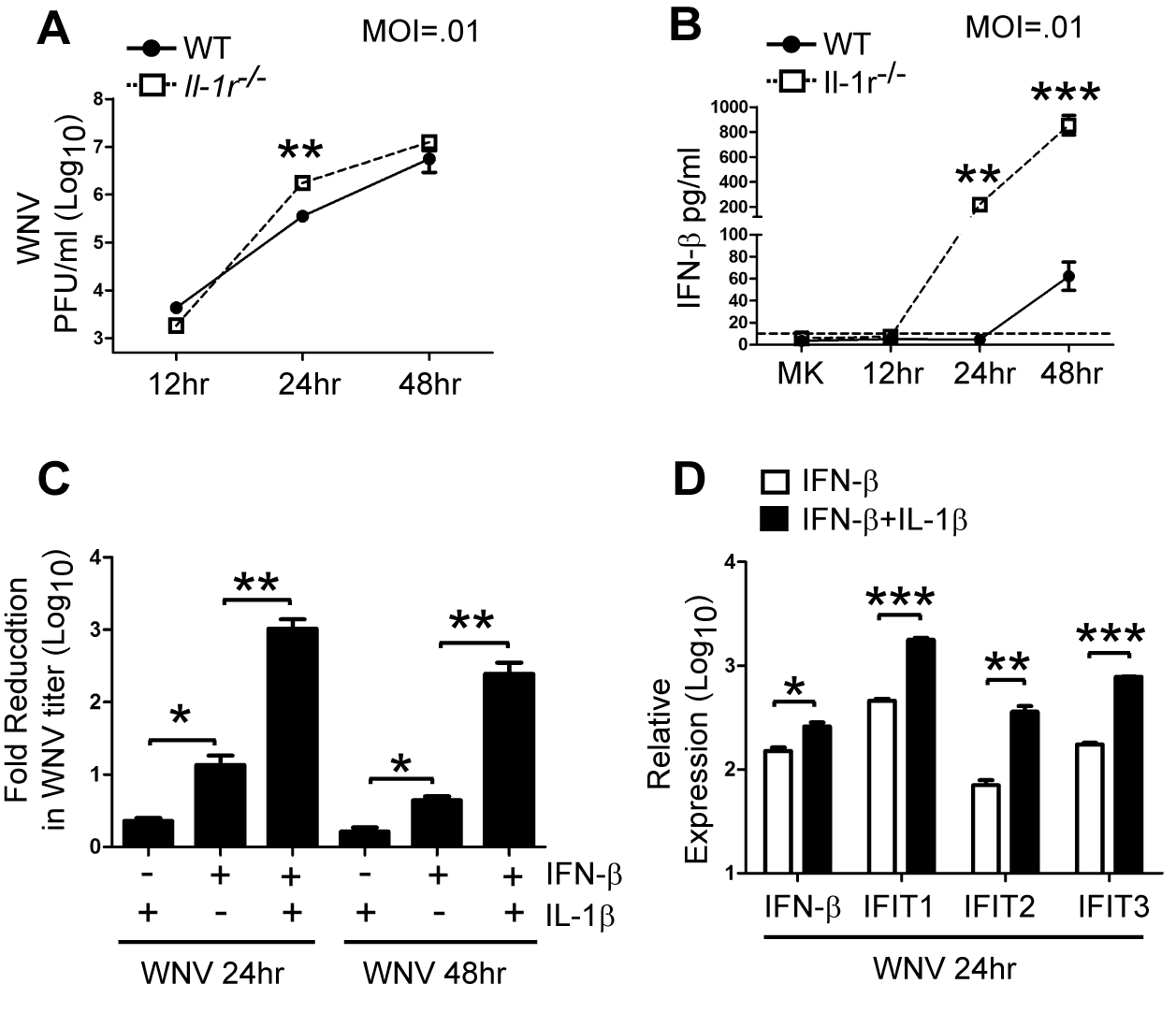

Supplement: Figure S6 — IL1β is associated with antiviral activity in the CNS. Cortical neurons were prepared from d15 embryos of WT (closed circles) or Il-1r−/− (open squares) animals and were assessed for viral load by plaque assay (A) or IFN-β (B) expression by ELISA after infection with low MOI (0.01) WNV-TX at 12, 24 and 48 hrs p.i. (C) The fold reduction in viral titer after IL-1β, IFN-β or IL-1β+IFN-β pre-treatment at 24 hr and 48 hr post infection with WNV-TX. ISG expression was assessed by quantitative Real-time PCR (qRT-PCR) using specific primers for IFIT1 (ISG56), IFIT2 (ISG54), IFIT3 (ISG49) and IFN-β made relative to GAPDH and normalized to mock values at 24 hr p.i. (D). Data are shown as Mean+/−S.E.M. for n = 3 per time-point and are representative of three independent experiments (A,B,D). For panel C, data are shown as Mean+/−S.E.M. for n = 9 and are compiled data from three independent experiments. * p<0.05, ** p<0.005, *** p<0.0005. Dashed line represents the lower limit of detection for each assay. (TIF) [file ppat.1003039.s006.tif]
