# Supplementary material for: IL-1β Signaling Promotes CNS-Intrinsic Immune Control of West Nile Virus Infection
Source: PLoS Pathog. 2012 Nov 29;8(11):e1003039. doi: 10.1371/journal.ppat.1003039 (PMC3510243; doi:10.1371/journal.ppat.1003039)
Supplement: Table S1 — The antibody response to West Nile virus is not altered in IL-1R or inflammasome deficient animals. Mice were infected with WNV and serum was isolated at days 6 or 8 p.i. from WT and inflammasome deficient animals. Serum IgG and IgM were detected by ELISA for antibody specific to WNV-E protein. For PRNT assay, serum was used to neutralize purified WNV-TX02 in BHK infections. Data is presented as the dilution at which antibody was detected at three standard deviations above mock or for PRNT, the dilution required to neutralize virus by 50%. (DOCX) [file ppat.1003039.s007.docx]

Table 1: Antibody responses to WNV are intact in the absence of inflammasome signaling

| **Antibody**  **Response** | **Day 6**  **WT** | **Day 6**  ***Il-1r^-/-^*** | **Day 8**  **WT** | **Day 8**  ***Il-1r^-/-^*** | **Day 8**  ***Casp-1^-/-^*** | **Day 8**  ***NLRP3^-/-^*** |
| --- | --- | --- | --- | --- | --- | --- |
| **IgM^a^** | 1620 | 1080 | 4242 | 5164 | 6604 | 7766 |
| **IgG^a^** | 40 | 20 | 8410 | 12611 | 11836 | 13580 |
| **PRNT_50_** | 1920 | 2560 | 6034 | 10094 | 9712 | 11946 |
| **Neutralization Index^b^** | 1.16 | 2.33 | .478 | .568 | .527 | .560 |

^a^IgM and IgG titers were determined by OD values that were 3 standard deviations above the mock controls.

^b^Neutralization index was calculated by dividing the PRNT50 by the total IgG titers at each time-point.
